# pH/ROS‐Responsive Injectable Hydrogel Co‐Loaded with B7‐H3 Blocker and NETs Suppressor Boosts OSCC Synergistic Immunotherapy

**DOI:** 10.1002/advs.202515431

**Published:** 2026-02-13

**Authors:** Huan Li, Yun Li, Yating Hu, Yanbing Yao, Yaodong He, Jing Li, Jiaqi Tang, Zhenyan Zhao, Yan Wang, Yulun He, Xiaolong Zhang, Xinjie Yang, Jianhua Wei

**Affiliations:** ^1^ State Key Laboratory of Oral and Maxillofacial Reconstruction and Regeneration, National Clinical Research Center for Oral Diseases, Shaanxi Key Laboratory of Stomatology, Department of Oral and Maxillofacial Surgery, School of Stomatology The Fourth Military Medical University Xi'an China

**Keywords:** B7‐H3, hydrogel, immunotherapy, neutrophil extracellular traps, oral squamous cell carcinoma

## Abstract

Oral squamous cell carcinoma (OSCC) demonstrates limited response to immunotherapies due to immunosuppression and metastasis. This study presents an injectable pH/ROS‐dual‐responsive hydrogel co‐loaded with enoblituzumab (B7‐H3 blocker) and Cl‐amidine (NETs suppressor). The hydrogel, formed by boronic ester bonds and Schiff base linkages, ensures precise drug release within the acidic, high‐ROS tumor microenvironment (TME). Notably, its localized intratumoral delivery avoids intravenous administration's systemic toxicity and off‐target effects, enabling high intratumoral accumulation with minimal systemic exposure. In orthotopic and subcutaneous OSCC models, intratumoral administration significantly suppressed tumor growth compared to monotherapies or controls. This enhanced antitumor effect arises from synergistic TME reprogramming: Cl‐amidine inhibits NETs formation to reduce barriers and facilitate robust CD4^+^/CD8^+^ T‐cell infiltration, while enoblituzumab, by targeting B7‐H3, restores cytotoxic T‐cell function and augments antibody‐dependent cellular cytotoxicity. Experimental evidence validates the suppression of OSCC invasion and metastasis through inhibition of B7‐H3 and NETs, which occurs by reversal of their induced epithelial‐mesenchymal transition (EMT). The hydrogel exhibits excellent biocompatibility without systemic toxicity. This TME‐responsive combination strategy offers a promising approach to enhance immunotherapy efficacy and overcome immune resistance in OSCC.

## Graphical abstract

A pH/ROS‐dual‐responsive injectable hydrogel, formed by dynamic boronic ester and Schiff base crosslinking, co‐delivers enoblituzumab (B7‐H3 blocker) and Cl‐amidine (NETs suppressor). Via localized intratumoral delivery, it synergistically reprograms the immunosuppressive tumor microenvironment, overcomes T cell infiltration barriers, restores cytotoxic T cell function, enhances OSCC immunotherapy efficacy, inhibits tumor metastasis, and minimizes systemic toxicity.

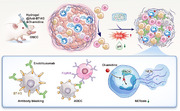

## 1 Introduction

Oral squamous cell carcinoma (OSCC) is the most prevalent malignant tumor in the oral‐maxillofacial area [1]. A significant number of patients are initially diagnosed with locally advanced stages, resulting in a poor 5‐year survival rate of 50%–60% and unfavorable prognosis [2]. Current standard treatment combines surgical resection with adjuvant chemoradiotherapy, yet frequently fails to restore function or improve long‐term survival [3]. In recent years, immunotherapy has emerged as an innovative therapeutic approach. Despite widespread clinical deployment of CTLA‐4/PD‐1 pathway‐blocking antibodies, significant therapeutic efficacy remains confined to patient subsets [4, 5], with the response rate often <20% [6, 7]. Thus, identifying novel immune escape molecules and elucidating their immunoregulatory mechanisms holds substantial clinical significance for improving patient outcomes.

B7 homolog 3, a key immune checkpoint protein overexpressed in multiple malignancies, correlates with poor prognosis in OSCC [8, 9]. Our previous research has established that B7‐H3 in OSCC cells facilitates immune escape by suppressing the activity of CD8^+^ T cells [10]. Enoblituzumab, a fully humanized anti‐B7‐H3 monoclonal antibody, features an engineered Fc domain that enhances CD16A binding and reduces CD32B engagement to augment antitumor efficacy [11]. However, its phase II trial (CP‐MGA271‐06) in recurrent/metastatic HNSCC was terminated prematurely due to safety concerns: 7 treatment‐related hemorrhagic deaths occurred among 62 patients receiving Enoblituzumab plus anti‐PD‐1 therapy. Recent clinical advances have renewed promise for OSCC immunotherapy: a 5‐year follow‐up of the phase III KEYNOTE‐048 trial confirmed that pembrolizumab with or without chemotherapy significantly improved overall survival in recurrent/metastatic HNSCC (including OSCC) [12]. Meanwhile, the phase I/II trial of YL201, a B7‐H3‐targeting ADC, has demonstrated promising efficacy in advanced solid tumors and a manageable safety profile, validating B7‐H3 as a viable therapeutic target [13]. While intravenous administration remains the primary delivery method for immunotherapy agents, ensuring rapid drug distribution and therapeutic onset, this approach frequently results in severe toxicities alongside poor tumor accumulation and off‐target effects [14, 15]. For instance, nivolumab plus ipilimumab therapy results in grade 3‐4 adverse events in 55% of patients and treatment discontinuation in 36% [16]. Localized immunotherapy presents an alternative approach, offering reduced systemic toxicity, lower dosing requirements, and enhanced bioavailability [17]. Injectable hydrogels have gained attention for their potential in minimally invasive local drug administration, yet traditional hydrogel‐based systems often rely on single‐stimulus responsiveness, rendering them inadequate for adapting to the dynamic tumor microenvironment [18]. We therefore engineered an injectable pH/ROS‐responsive hydrogel for intratumoral delivery. This carrier specifically degrades in acidic, high‐ROS tumor microenvironments, enabling targeted drug release that maximizes tumor‐site concentration while minimizing systemic exposure. The pH/ROS responsiveness, achieved through Schiff base bonds and phenylboronic acid ester bonds, enables precise recognition of tumor microenvironment characteristics, thereby enhancing therapeutic specificity and safety [19].

Neutrophil infiltration density within the tumor microenvironment demonstrates a strong correlation with tumor progression and immune escape [20, 21]. Activated neutrophils release abundant neutrophil extracellular traps (NETs), which trap CD8^+^ T cells within the tumor stroma and prevent them from infiltrating into tumors [22]. NETs consist of network structures containing DNA, histones, and antimicrobial proteins [23]. These structures create a physical obstruction that not only limits immune cell penetration into the TME but also suppresses T cell function [24, 25]. Cl‐amidine hydrochloride, an orally administered pan‐PAD inhibitor targeting PAD1, PAD3, and PAD4, suppresses NETosis by blocking PAD4‐mediated histone citrullination in neutrophils [26, 27]. Therefore, we engineered an injectable pH/ROS‐dual‐responsive hydrogel for the co‐delivery of a B7‐H3 blocking antibody and the NETs inhibitor Cl‐amidine hydrochloride. The pH/ROS responsiveness facilitates targeted drug release in the TME, which exhibits low pH and high ROS levels [28], reducing systemic side effects and optimizing therapeutic efficacy. This platform enables TME‐triggered drug release to achieve two synergistic objectives: inhibiting NETs formation to facilitate immune cell infiltration into tumor cores while simultaneously blocking B7‐H3 to enhance cytotoxic T cell activity, thereby promoting effective immune‐mediated tumor clearance.

In summary, this study presents the first demonstration of a dual‐responsive hydrogel combining B7‐H3 checkpoint blockade with NETs suppression. This material‐immunotherapy integration overcomes limitations of conventional therapies by enhancing antitumor efficacy while reducing systemic toxicity, offering a new paradigm for OSCC immunotherapy.

## 2 Materials and Methods

### 2.1 Clinical Samples

OSCC tissues and adjacent normal tissues were procured from 30 patients at the Stomatological Hospital of the Fourth Military Medical University between 2023 and 2024. None of the enrolled patients underwent preoperative radiotherapy or chemotherapy. The study received ethical clearance from the Medical Research Ethics Committee of the Fourth Military Medical University (ethical approval No.: KQ‐YJ‐2023‐031), and all participants provided informed consent.

### 2.2 Cell Lines and Isolation

CAL‐27 (RRID: CVCL_1107) was obtained from the Peking University School of Stomatology. SCC‐9 (RRID: CVCL_1685) was initially purchased from the ATCC. All cell lines used in the experiment were all authenticated by short tandem repeat (STR) profiling. CAL‐27 and SCC‐9 cells were cultured in RPMI‐1640 medium (Gibco, USA) supplemented with 10% FBS and 1% penicillin‐streptomycin (Gibco, USA), and maintained in a humidified incubator with 5% CO_2_ at 37°C. Preparation of human PBMCs and isolation of CD8^+^ T cells were as described in our previously published articles, with the reagents used and procedures consistent with those mentioned therein [10]. Primary human neutrophils were isolated from the peripheral blood of healthy donors following the manufacturer's protocol (#P9040, Solarbio, China).

### 2.3 Bioinformatic Analysis

Both pan‐cancer expression of B7‐H3 and survival analysis in HNSC patients were analyzed using The Cancer Genome Atlas (TCGA) database via the GEPIA2 platform (http://gepia2.cancer‐pku.cn/#index) [29]. Spearman's rank correlation analysis, performed via the TISIDB platform, was used to assess associations between B7‐H3 expression and the infiltration of T cells and neutrophils in HNSCC (http://cis.hku.hk/TISIDB/) [30]. Correlations between B7‐H3/NETs expression and EMT markers were analyzed using Spearman's rank correlation (TIMER2.0, http://timer.cistrome.org/) [31].

### 2.4 Plasmid Transfection

OSCC cells (CAL‐27, SCC‐9) were subjected to transfection with B7‐H3‐overexpressing plasmids (OE‐B7‐H3) or B7‐H3‐targeting shRNA plasmids (Sh‐B7‐H3) using Lipofectamine 2000 (Invitrogen) following standard protocols. The human B7‐H3 overexpression plasmid was constructed using pcDNA3.1 as the vector, with the full‐length CDS sequence of B7‐H3 inserted (NCBI: NM_025240, 951bp, GenePharma, Shanghai, China), while the target sequence of Sh‐B7‐H3 was GCAGCTGACAGATACCAAACA. Co‐transfection of OE‐B7‐H3 and siB7‐H3 was performed using Lipofectamine 2000 (Invitrogen) according to the reagent's instructions. The B7‐H3 siRNA sequences were: Guide strand 5’‐ACUUAGAGAACUAAGGUUGUG‐3’, Passenger strand 5’‐CAACCUUAGUUCUCUAAGUCA‐3’ (GenePharma). The negative control siRNA (siNC) sequences are as follows: Guide strand 5’‐ACGUGACACGUUCGGAGAATT‐3’, Passenger strand 5’‐UUCUCCGAACGUGUCACGUTT‐3’ (GenePharma). Twenty‐four hours post‐transfection, the PI3K inhibitor LY294002 (HY‐10108, 20µM, MCE) was added to the cell culture system.

### 2.5 NETs Induction

To induce NETs formation, human neutrophils (1 × 10^6^ cells/mL) were incubated with phorbol 12‐myristate 13‐acetate (100 nM, PMA, #HY‐18739, USA) for 4 h. The concentration of NETs was determined with a Quant‐iT PicoGreen dsDNA assay kit (#P7581, Invitrogen). Low and high concentrations of NETs were defined as 0.25 and 0.5 µg/mL, respectively.

### 2.6 Scratch Wound Healing Assay

OSCC cells (CAL‐27, SCC‐9) were plated in 6‐well plates at a density of 1 × 10^6^ cells per well and cultured in serum‐free medium. Each well was scratched with a sterile 200 µL pipette tip. Wound closure was photographed at 0 and 24 h and quantified using ImageJ.

### 2.7 Migration and Invasion Assay

For migration and invasion assays, OSCC cells (CAL‐27, SCC‐9, 5 × 10^4^) were plated into transwell inserts (8 µm, Corning) containing 200 µL of RPMI‐1640 (serum‐free), with migration assays using inserts placed in 24‐well plates and invasion assays using inserts pre‐coated with Matrigel. After 24 h culture, residual cells in the upper chamber were wiped away with cotton swabs. Cells were fixed with 4% formaldehyde, stained with 1% crystal violet, and the numbers of membrane‐traversing cells were counted in 3 random fields.

### 2.8 T Cell Cytotoxicity Test

At a 1:10 ratio, CAL‐27 cells and CD8^+^ T cells were co‐cultured with or without NETs. Cytotoxicity was assessed with a CCK‐8 kit, and the assay was performed following standard protocols [10]. The OD value was measured at 450 nm. Cytotoxicity% = (OD_control_ − OD_sample_)/OD_control_ × 100%. The OD_control_ value represents CAL‐27 cells cultured alone without CD8^+^ T cells or NETs.

### 2.9 ELISA

We used human ELISA kits (Westang Bio‐Tech Co., Ltd., Shanghai, China) to detect TNF‐α, IFN‐γ, perforin, and granzyme B levels in CD8^+^ T cells co‐cultured with High‐NETs (0.5 µg/mL) or Low‐NETs (0.25 µg/mL) to evaluate the impact of NETs on CD8^+^ T cell immune activity. Sample absorbance was determined at 450 nm, and concentrations were derived from a standardized curve.

### 2.10 Synthesis of Hydrogel@Anti‐B7‐H3&Cl‐amidine

Phenylboronic acid‐grafted oxidized dextran (POD) was synthesized through the following procedure: First, oxidized dextran (OD) was prepared by fully dissolving sodium periodate (#7790‐28‐5, Sigma–Aldrich) and dextran (#9004‐54‐0, Sigma–Aldrich) in deionized water. Sodium periodate was added to the dextran solution, with the mixture reacting in the dark for 24 h. The product was dialyzed against deionized water using a 14 kDa‐cutoff membrane for 3 days, then lyophilized to yield OD. Next, the resulting OD was dissolved in deionized water, and phenylboronic acid was introduced. The mixture was stirred overnight to facilitate the grafting reaction. Finally, the product was dialyzed again with a 3.5 kDa‐cutoff membrane for 3 days and lyophilized to yield POD. Catechol‐modified quaternary ammonium chitosan (CQCS) was synthesized through the following procedure: Quaternary ammonium chitosan was fully dissolved in deionized water, followed by the addition of 1‐ethyl‐3‐(3‐dimethylaminopropyl) carbodiimide (#25952‐53‐8, EDC, Sigma–Aldrich) and N‐hydroxysuccinimide (#6066‐82‐6, NHS, Sigma–Aldrich) to activate the solution for a period of 30 min. Subsequently, 3,4‐dihydroxyphenylacetic acid (#102‐32‐9, Sigma–Aldrich) was introduced into the mixture. The pH was adjusted to 5‐6 to maintain optimal reaction conditions. The product was then dialyzed against deionized water in a 14 kDa‐cutoff bag for 2 days and subsequently lyophilized to obtain CQCS. POD and CQCS were synthesized by Xi'an Ruixi Biological Technology Co., Ltd. according to previously described methods. Cl‐amidine (5 mg/mL, #HY‐100574A, MCE) and POD were co‐dissolved in deionized water to prepare a POD solution containing Cl‐amidine, while Enoblituzumab (5 mg/mL, #HY‐P9966, MCE) was dissolved in a CQCS solution to prepare an antibody‐loaded CQCS solution. Then, the two solutions were combined in a 1:1 volume ratio, rapidly stirred, and allowed to stand for 3 min to form a hydrogel via dynamic boronic ester and Schiff base linkages.

### 2.11 Physicochemical Characterization

Prepared hydrogel samples, after freeze‐drying and sputter‐coating with gold to enhance conductivity, were observed for microscopic morphology via scanning electron microscopy (SEM, Sigma 300, Germany). Prepared hydrogel samples were immersed in pH 5 PBS and 1 mm H_2_O_2_ ultrapure water, with changes observed at different intervals. Two samples were prepared, one side of each contacted. After a period, one end was lifted to observe self‐healing. Hydrogels were shaped into cylinders (35 mm diameter, 3 mm thickness), and rheological tests (parallel‐plate rheometer, MARS40, Haake, Germany) yielded modulus results at different frequencies. The swelling ratio of hydrogels was determined by weighing samples before immersion in PBS, removing surface liquid with filter paper at regular intervals, reweighing, and calculating using the formula: Swelling ratio = (Ms ‐ Md)/Md (Ms: post‐swelling weight, Md: pre‐swelling weight). Drug‐loaded hydrogels were immersed in solutions with varying pH/H_2_O_2_ levels. Released Cl‐amidine and enoblituzumab were quantified via UV absorption and BCA Protein Assay Kit (#P0010S, Beyotime, China), respectively. Cl‐amidine standard curve: *y* = 0.027*x* ‐ 0.0007 (R^2^ = 0.9998; *x*: absorbance, *y*: concentration).

### 2.12 Comparison of Hydrogel Systems and Pharmacokinetic Study

Three hydrogels were prepared according to their respective kit protocols: (a) ROS‐Responsive Quaternary Ammonium Salt Tannic Acid–Chitosan Hydrogel: This formulation was prepared by rapidly mixing Component A (a chitosan–tannic acid solution) with Component B (NaHCO_3_ solution), resulting in gelation within 30 s. (b) pH‐Responsive Polyethylene Glycol/Carboxymethyl Chitosan Hydrogel: This hydrogel was formed by mixing Component A (a PEG solution) with Component B (a carboxymethyl chitosan solution), with gelation occurring progressively over approximately 20 min. (c) Adhesive Catechol–Chitosan Hydrogel (non‐stimuli‐responsive): This formulation was prepared by first mixing Component A (a catechol–chitosan solution) with Component B (H_2_O_2_), followed by the addition of Component C (HRP). The mixture was allowed to stand for 10 min to form a stable gel. Cl‐amidine (5 wt%) and Enoblituzumab (2 wt%) were pre‐mixed into Component A of each hydrogel for drug loading. The in vitro drug release assay was conducted as previously described.

For the in vivo pharmacokinetic study, three groups (n = 3) were established: (a) Blank control group; (b) Intratumoral dual‐drug group (intratumoral injection of drug‐loaded hydrogel); (c) Intravenous dual‐drug group (intravenous injection of dual‐drug solution). Tumor tissues and serum samples were collected at various time points. Cl‐amidine concentration was quantified using LC‐MS/MS (UltiMate 3000 RS system, Thermo Fisher Scientific Co., Ltd.). Enoblituzumab concentration was determined with a Human IgG ELISA Kit (Beyotime, Cat. PI484) according to the manufacturer's instructions.

### 2.13 In Vivo Animal Therapeutic Experiments

NOD.Cg‐*Prkdc^scid^Il2rg^em1Smoc^* (M‐NSG) mice (female, ∼20 g, 4–6 weeks) were obtained from Model Organisms Center (Shanghai, China). All mice were housed in a specific pathogen‐free (SPF) environment with standard husbandry conditions. To evaluate immune reconstitution efficiency from different donors, three healthy donors were selected. Human peripheral blood mononuclear cells (PBMCs) and neutrophils were co‐injected intravenously into M‐NSG mice at a dose of 5 × 10^6^ cells per mouse (n = 3 mice per donor), establishing a Hu‐PBMC+NE reconstruction mouse model [32]. Body weight changes were monitored every 5 days for 30 days post‐injection to assess general health status. At 2 weeks post‐injection, peripheral blood from mice was collected and processed into single‐cell suspensions, which were then stained with fluorochrome‐conjugated antibodies: anti‐hCD45‐PE (#PE‐65109, Proteintech, Wuhan, China), anti‐hCD3‐FITC (#300305, BioLegend, San Diego, USA), and anti‐hCD66b‐FITC (#305103, BioLegend, San Diego, USA).

OSCC cells (CAL‐27, 1 × 10^7^) were injected into the tongue submucosa of Hu‐PBMC+NE mice, and once tumors reached ∼50 mm^3^, the mice were randomized into 5 groups (n = 5): Normal Saline, Hydrogel, Hydrogel@Cl‐amidine, Hydrogel@Anti‐B7‐H3, and Hydrogel@Anti‐B7‐H3&Cl‐amidine (intratumoral injection, BD Precision Glide needle, 20 µL per injection, 4 injections were given, administered every 2 days). Isotype control experiments used hydrogels loaded with Human IgG1 kappa (#HY‐P99001, MCE) at the same loading concentration as Enoblituzumab. Subcutaneously injecting OSCC cells (CAL‐27, 1 × 10^7^) into the right dorsal flank of Hu‐PBMC+NE mice, the animals were randomized into 5 groups (n = 3) once tumors reached ∼50 mm^3^ (intratumoral injection, BD Precision Glide needle, 20 µL per injection, 6 injections in total, administered every 3 days). After tail vein injection of OSCC cells (CAL‐27, SCC‐9, 2 × 10^6^), the mice were randomly assigned to the following treatment groups: Blank, Normal Saline, Sh‐B7‐H3, OE‐B7‐H3, Cl‐amidine (5 mg/kg, q3d, i.v.), PMA (0.1 mg/kg, q3d, i.v.), NETs (5 µg per mouse, i.v.), PMA + Cl‐amidine, and NETs + DNase 1(DNase 1, 5 mg/kg, i.v., #HY‐108882, MCE, USA). Corresponding treatments were administered according to group assignments. And lung metastases were analyzed at 20 days post‐injection. Tumor volume (V) = (L × W^2^)/2. Bioluminescence imaging via IVIS Spectrum (PerkinElmer, USA) followed i.p. injection of D‐luciferin (150 mg/kg, #HY‐12591B, MCE). At the end of the experiment, mice were euthanized, and tumors/lungs were harvested for histology. All mice were handled in accordance with protocols approved by the Committee on the Use of Live Animals in Teaching and Research of the Fourth Military Medical University (ethical approval No.: KQ‐2023‐012).

### 2.14 Western Blot Analysis

Total protein from each sample was isolated and analyzed via Western blot following established protocols. Protein signals were detected with an enhanced chemiluminescence kit (Thermo Fisher Scientific, USA). Primary antibodies were anti‐CD16A (#GB11406, 1:500, Servicebio) and anti‐CD32B (#ab68423, 1:5000, Abcam), with the secondary antibody being HRP‐conjugated goat anti‐rabbit IgG (H&L) (#ab6721, 1:2000, Abcam).

### 2.15 Flow‐cytometry assay

Flow cytometry was used to evaluate the expression levels of granzyme B, TNF‐α, perforin, and IFN‐γ in CD8^+^ T cells isolated from tumor tissues of each treatment group. After sorting, these CD8^+^ T cells were labeled with anti‐granzyme B‐PE (#12‐8896‐42, Invitrogen), anti‐IFNγ‐PE (#12‐7319‐41, Invitrogen), anti‐perforin‐FITC (#11‐9994‐42, Invitrogen), and anti‐TNF‐α‐FITC (#11‐7349‐81, Invitrogen). Briefly, cells were gated on single, live cells, then on CD3^+^ cells, and subsequently on CD8^+^ T cells for analysis of TNF‐α, IFN‐γ, perforin, and granzyme B expression.

### 2.16 Histology and Immunofluorescence

For IHC staining, primary antibody incubation was conducted using anti‐B7‐H3 (#ab227670, Abcam, UK) at a dilution of 1:100 for 10 min at room temperature. IHC staining was scored by two pathologists based on positive rate, with IHC‐score = (1× Weak %) + (2× Moderate %) + (3× Strong %). IF staining of tumor sections were incubated with primary antibodies overnight at 4°C, including anti‐B7‐H3 (#ab227679, 1:100, Abcam), anti‐CitH3 (#ab281584, 1:2000, Abcam), anti‐MPO (#GB11224, 1:1000, Servicebio), anti‐CD4 (#67786‐1‐Ig, 1:200, Proteintech), anti‐CD8 (#GB12068, 1:2000, Servicebio), anti‐CD16 (#ab246222, 1:100, Abcam), anti‐human PD‐1 (#GB12338, 1:1000, Servicebio), anti‐human CTLA‐4 (#702534, 2µg/mL, Invitrogen), anti‐human TIM‐3 (#MA5‐32841, 1:100, Invitrogen), and anti‐human LAG‐3 (#MA5‐44249, 1:200, Invitrogen). Antibodies for EMT detection in OSCC cell (CAL‐27, SCC‐9) coverslips and tumor tissues included anti‐E‐cadherin (#GB11082, 1:200) and anti‐N‐cadherin (#GB12135, 1:500), both from Servicebio. Anti‐Ki67 (#GB111499, 1:1000, Servicebio) was used for detecting tumor proliferation via immunofluorescence. The TUNEL kit (#G1504, Servicebio) was used for detection. The positive cells were quantified using ImageJ.

### 2.17 Safety Evaluation

Healthy mice (BALB/c, n = 3/group) were treated with hydrogel formulations. At 2 weeks post‐treatment, major organs, including heart, liver, kidney, lung, and spleen were collected for HE staining. Serum was collected for biochemical analysis using an automatic biochemistry analyzer (Chemray 240, Shenzhen Rayto Life Technology, China).

### 2.18 Statistical Analysis

All analyses used SPSS 24.0 (IBM, USA) and GraphPad Prism V8.0 (GraphPad Software, USA). Randomization was applied for animal grouping using a computer‐generated randomization list to ensure baseline consistency. Blinding was implemented: single‐blinding for tumor volume measurement and histochemical staining quantification in animal experiments (operators unaware of group assignments), and double‐blinding for IHC scoring of clinical specimens (two pathologists unaware of sample sources). Sample size was determined based on prior OSCC immunological research and power analysis. The sample size (n) for each experiment is indicated in the corresponding figure legend or method description. Prior to analysis, data were tested for normality and variance homogeneity. For normally distributed data, intergroup differences were assessed via independent samples t‐test (two groups) or one‐way ANOVA with Tukey's post hoc test (multiple groups). Notably, the Tukey's post hoc test inherently controls for type I error in multiple comparisons. Measurement data are presented as mean ± SD, with repeated measures ANOVA used for multi‐timepoint data. Survival was analyzed via Kaplan‐Meier, with curves compared using log‐rank test. Pearson's correlation coefficient was used for correlations between continuous variables, while Spearman's rank correlation was used for those from bioinformatic databases. All in vitro experiments were independently repeated at least three times to ensure reproducibility. *p* < 0.05 was considered statistically significant.

## 3 Results

### 3.1 B7‐H3 Overexpression and NETs Accumulation Drive Immunosuppression by Inhibiting T Cell Infiltration and Function in OSCC

**FIGURE 1:**
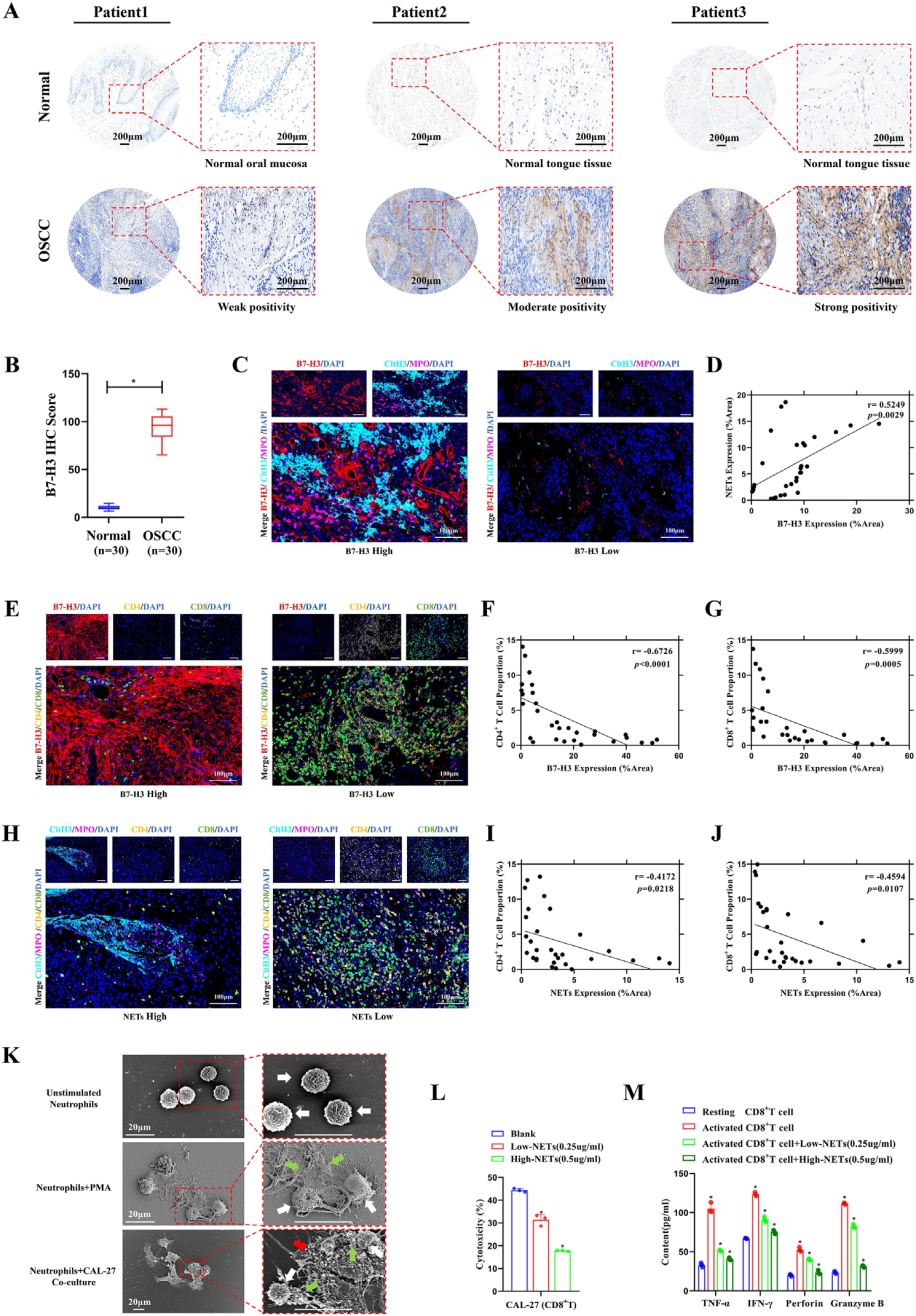
Clinical Correlates of B7‐H3/NETs‐Driven Immunosuppression in OSCC. (A) Representative IHC images of B7‐H3 expression in three paired OSCC and adjacent normal tissues from different patients: Patient 1: Weak B7‐H3 positivity in OSCC tissue; Patient 2: Moderate B7‐H3 positivity in OSCC tissue; Patient 3: Strong B7‐H3 positivity in OSCC tissue (Scale bars: 200 µm). (B) Comparison of IHC scores for B7‐H3 expression between tumor tissues and adjacent normal tissues from 30 patients (^*^ *p* < 0.05). (C) Representative IF Staining of B7‐H3 and NETs (CitH3^+^/MPO^+^) in Clinical OSCC Specimens. (D) Scatter Plot for Pearson's Correlation Analysis of B7‐H3 and NETs Expression in OSCC (r = 0.5249, *p* = 0.0029). (E) Representative IF Staining of B7 ‐ H3, CD4^+^, and CD8^+^ T Cells in Clinical OSCC Specimens. (F) Scatter Plot for Pearson's Correlation Analysis of B7‐H3 and CD4^+^ T Cell Expression in OSCC (r = −0.6726, *p* < 0.0001). (G) Correlation Analysis of B7‐H3 and CD8^+^ T Cell Expression in OSCC (r = −0.5999, *p* = 0.0005). (H) Representative IF Staining of NETs (CitH3^+^/MPO^+^), CD4^+^, and CD8^+^ T Cells in Clinical OSCC Specimens. (I) Scatter Plot for Pearson's Correlation Analysis of NETs and CD4^+^ T Cell Expression in OSCC (r = −0.4172, *p* = 0.0218). (J) Correlation Analysis of NETs and CD8^+^ T Cell Expression in OSCC (r = −0.4594, *p* = 0.0107). (K) SEM Characterization of Surface Morphology and Interaction Between NETs and Tumor Cells in Co‐Culture (White arrows: Neutrophils; Green arrows: NETs; Red arrows: CAL‐27 cells; scale bars: 20 µm;). (L) High NETs Concentrations Inhibit CD8^+^ T Cell Cytotoxicity in CCK8 Assay. (M) ELISA Detection of Cytokine Expression Levels in CD8^+^ T Cells Treated with NETs Suspensions. NETs were induced by incubating neutrophils with 100 nm PMA for 4 h; high/low NETs concentrations were 0.5/0.25 µg/mL. Clinical samples: n = 30; in vitro experiments: n = 3. Statistical methods: t‐test for two groups, ANOVA‐Tukey for multiple groups, and Pearson's correlation analysis for correlation assays. Data are presented as mean ± standard deviation (^*^ *p* < 0.05).

IHC staining for B7‐H3 in OSCC and adjacent normal tissues from 30 patients revealed significantly higher B7‐H3 expression in OSCC tissues compared with normal tissues. Figure 1A displays representative images of three patients, with B7‐H3 expression in OSCC tissues showing weak positivity, moderate positivity, and strong positivity, respectively. Compared with adjacent normal tissues, quantitative analysis of IHC scores from 30 patients revealed significantly higher average B7‐H3 IHC scores in OSCC (Figure 1B, *p* < 0.05). Pan‐cancer analysis revealed consistent high expression of B7‐H3 across human malignancies, including DLBC, GBM, HNSC, KIRC, ESCA, COAD, CHOL (Figure S1A). In HNSC patients, survival analysis showed high B7‐H3 expression was strongly linked to worse overall survival (OS) (Figure S1B, *p* < 0.05). These results indicate B7‐H3 upregulation could be a prognostic indicator and underline its potential as a treatment target in aggressive tumors.

Representative immunofluorescence (IF) staining of clinical OSCC specimens showed a positive correlation between B7‐H3 and neutrophil extracellular traps (NETs, CitH3^+^/MPO^+^) expression, with increased B7‐H3 signal intensity accompanying elevated NETs formation (Figure 1C). Pearson's correlation analysis of 30 paired OSCC specimens demonstrated a significant positive correlation between B7‐H3 and NETs expression (*p* = 0.0029, r = 0.5249, Figure 1D), indicating that B7‐H3 upregulation is accompanied by enhanced NETs formation in OSCC. Similarly, bioinformatics analysis demonstrated a positive correlation between B7‐H3 expression and neutrophil infiltration (*p* = 0.0462, rho = 0.087, Figure S1E). In the co‐culture system (Figure S4A,C), neutrophils co‐cultured with CAL‐27 cells overexpressing B7‐H3 (OE‐B7‐H3) showed a significant increase in NETs density compared with the OE‐NC control group (^*^ *p* < 0.05). Conversely, knockdown of B7‐H3 (Sh‐B7‐H3) in CAL‐27 cells substantially reduced NETs formation in co‐cultured neutrophils relative to the Sh‐NC group (^*^ *p* < 0.05). In parallel experiments using recombinant protein stimulation (Figure S4B,D), treatment of neutrophils with recombinant B7‐H3 protein (0.5 µg/mL) markedly enhanced NETs density (^*^ *p* < 0.05) ‐ an effect comparable to that induced by the positive control PMA (100 nM). Together, these findings demonstrate that B7‐H3, whether expressed by OSCC cells or administered as a recombinant protein, directly promotes NETs formation in human neutrophils.

IF staining showed a negative correlation between B7‐H3 expression and T cells (CD4^+^, CD8^+^) infiltration: high B7‐H3 regions showed decreased CD4^+^/CD8^+^ T cell accumulation, whereas low B7‐H3 areas exhibited significantly enhanced T cell presence (Figure 1E). Pearson's correlation analysis of 30 OSCC patient samples confirmed a significant inverse correlation between B7‐H3 and CD4^+^ T cell expression (*p* < 0.0001, r = −0.6726, Figure 1F) and with CD8^+^ T cell expression (*p* = 0.0005, r = −0.5999, Figure 1G). Similarly, bioinformatics analysis showed significant negative correlations of B7‐H3 expression with CD8^+^ T cell infiltration (*p* = 0.0025, rho = −0.132, Figure S1C) and CD4^+^ T cell infiltration (*p* = 0.000114, rho = −0.168, Figure S1D). In regions with abundant NETs (CitH3^+^/MPO^+^), T cell (CD4^+^, CD8^+^) infiltration was reduced, whereas NETs deficient regions exhibited significantly increased T cell accumulation (Figure 1H). Correlation analysis revealed inverse relationships between NETs and CD4^+^ T cell (*p* = 0.0218, r = −0.4172, Figure 1I) as well as CD8^+^ T cell (*p* = 0.0107, r = −0.4594, Figure 1J). These results indicate that B7‐H3 and NETs may suppress T cell infiltration, thereby promoting immunosuppression in the tumor microenvironment.

Scanning electron microscopy (SEM) observations revealed that resting neutrophils exhibited a round morphology (white arrows). Following stimulation with 100 nM PMA for 4 h, neutrophils generated extracellular NETs characterized by web‐like structures (green arrows). When co‐cultured with CAL‐27 cells, the generated NETs directly interacted with tumor cells (red arrows), forming a dense reticular network that surrounded the CAL‐27 cells (Figure 1K). In CD8^+^ T cell cytotoxicity assays, high‐concentration NETs markedly reduced CD8^+^ T cell cytotoxicity against CAL‐27 cells (Figure 1L, *p* < 0.05). The ELISA results showed that CD8^+^ T cells treated with NETs exhibited lower levels of TNF‐α, IFN‐γ, perforin, and granzyme B, with the secretion of these activity factors decreasing as the concentration of NETs increased (Figure 1M, *p* < 0.05), suggesting that NETs may suppress CD8^+^ T cell function.

### 3.2 Design, Preparation, and Physicochemical Characterization of Hydrogel@Anti‐B7‐H3&Cl‐amidine

**FIGURE 2:**
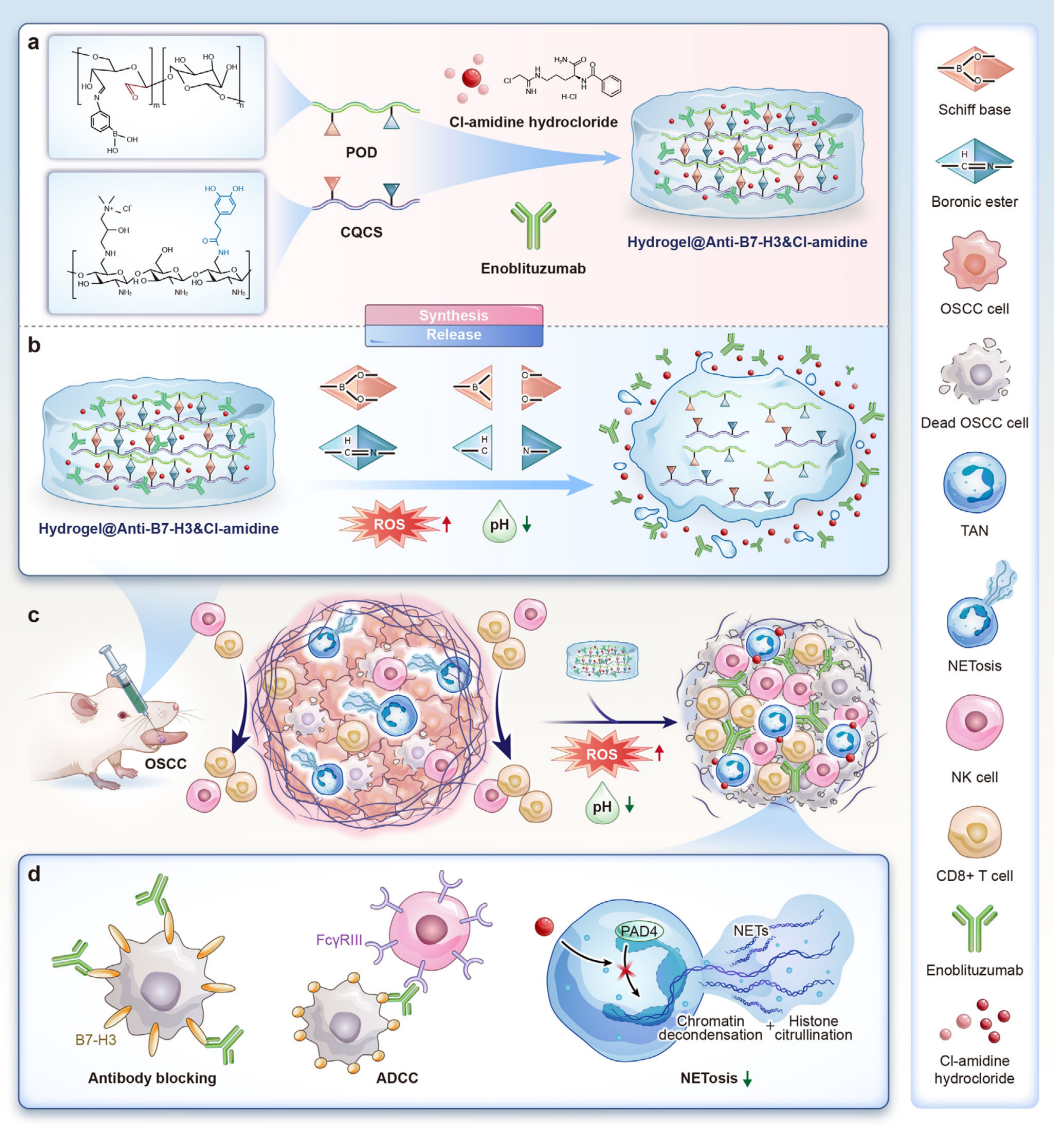
Schematic of tumor microenvironment‐responsive dual‐drug‐loaded injectable Hydrogel@Anti‐B7‐H3&Cl‐amidine for OSCC therapy. (a) Synthesis schematic of Hydrogel@Anti‐B7‐H3&Cl‐amidine. The hydrogel was formed via crosslinking of CQCS and POD through dynamic boronic ester and Schiff base bonds. (b) Drug release mechanism of Hydrogel@Anti‐B7‐H3&Cl‐amidine. (c) Intratumoral administration schematic of the hydrogel. (d) The hydrogel's molecular mechanism includes B7‐H3 blockade to reactivate CD8^+^ T cells, ADCC enhancement, and PAD4 inhibition to suppress NETs formation, collectively mediating antitumor effects.

The strategic design of Hydrogel@Anti‐B7‐H3&Cl‐amidine for OSCC therapy is illustrated in Figure 2. The hydrogel formed by crosslinking CQCS and POD via dynamic boronic ester and Schiff base bonds, with pH/ROS dual responsiveness. The synthesis schematic (Figure 2a) shows that enoblituzumab and Cl‐amidine were loaded into a pH/ROS‐responsive injectable hydrogel matrix, enabling dual targeting of B7‐H3 and NETs. The drug release mechanism (Figure 2b) is tailored for TME responsiveness, where pH/ROS‐sensitive matrix degradation enables the sustained release of enoblituzumab and Cl‐amidine. Leveraging the acidic TME, hydrogel pH responsiveness stems from dynamic Schiff base linkages, whereby TME acidity promotes their hydrolytic cleavage to induce matrix swelling and permeability changes. In contrast, neutral/alkaline pH stabilizes these linkages to mitigate swelling. Concurrently, phenylboronic ester moieties respond to elevated TME ROS through oxidation‐driven hydrophilic‐to‐hydrophobic transitions in the polymer network, disrupting hydrogel architecture to facilitate controlled disassembly and triggered drug release. This dual‐responsive design enables precise cargo delivery tailored to the acidic and hyper‐ROS tumor microenvironment. Figure 2c demonstrates the intratumoral administration of the hydrogel in OSCC models. Following administration, the hydrogel responds to TME's low pH and high ROS, triggering controlled drug release and reprogramming its immunosuppressive state. As a PAD4 inhibitor, Cl‐amidine suppresses NETs formation, allowing massive immune cell infiltration into the TME to exert anti‐tumor effects. Concurrently, enoblituzumab (anti‐B7‐H3) mediates B7‐H3 blockade to reactivate CD8^+^ T cells and enhance antibody‐dependent cellular cytotoxicity (ADCC), as illustrated in Figure 2d. This innovative material design and administration strategy address the challenges of systemic toxicity and poor efficacy associated with monoclonal antibody monotherapy, achieving synergistic tumor suppression through TME reprogramming.

**FIGURE 3:**
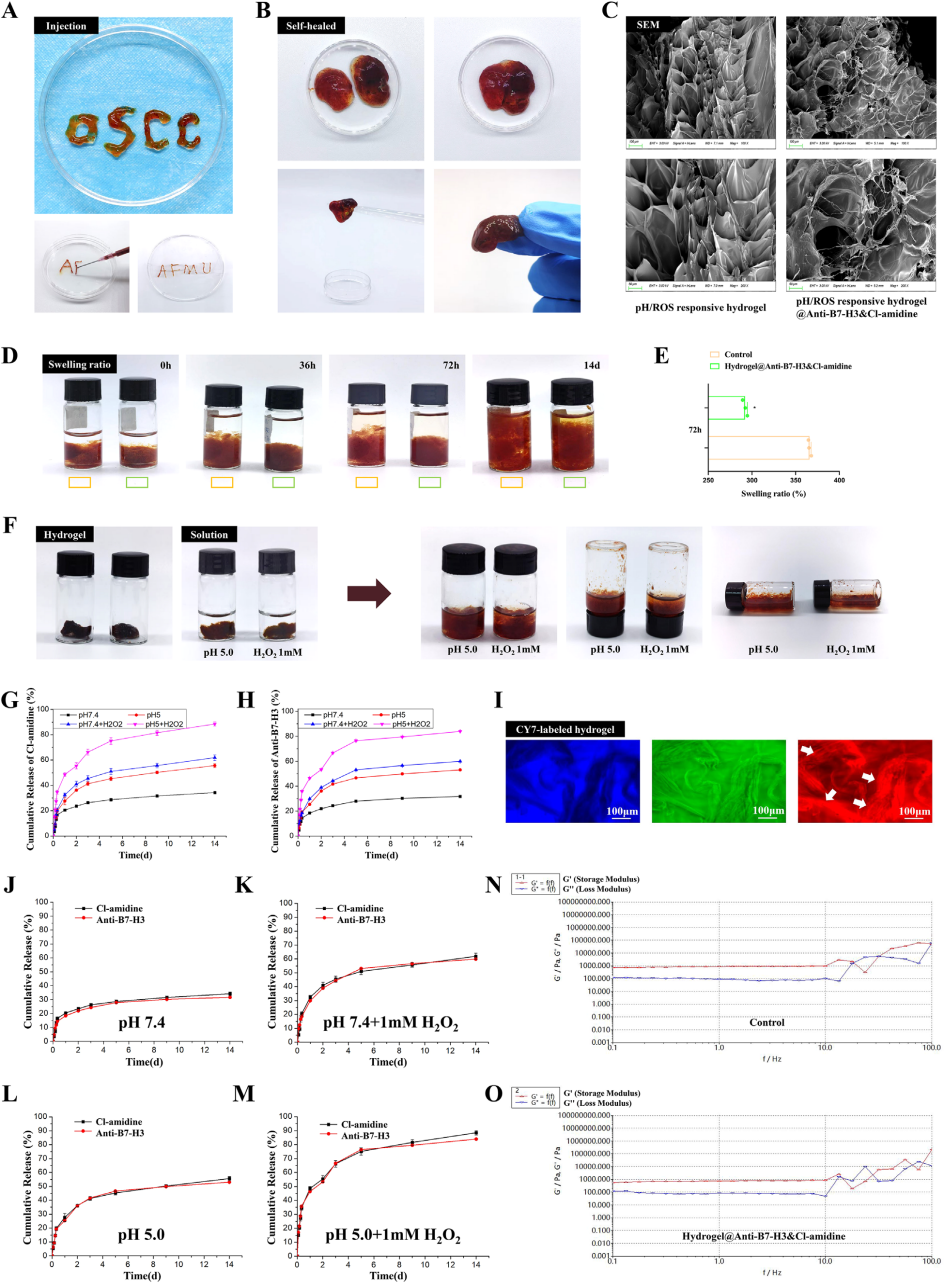
Physicochemical characterization and TME‐responsive drug release of dual‐drug‐loaded injectable Hydrogel@Anti‐B7‐H3&Cl‐amidine. (A) Injectability demonstration of the hydrogel. (B) Self‐healing property of the hydrogel. (C) SEM images of the hydrogel before and after drug loading. (D) Swelling ratio and 14‐day degradation images of blank and drug‐loaded hydrogels. (E) Statistical analysis of swelling ratio at 72 h. (F) pH/ROS‐responsive characterization. (G–H) Single‐drug release kinetics of Cl‐amidine (G) and Anti‐B7‐H3 (H). (I) CY7‐labeled hydrogel showing grid structure (white arrows) under red light excitation. (J–M) Drug release was tested in media mimicking TME and physiological conditions. Dual‐drug release profiles in (J) pH 7.4 PBS, (K) pH 7.4 PBS + 1 mm H_2_O_2_, (L) pH 5.0 PBS, and (M) pH 5.0 PBS + 1 mm H_2_O_2_. (N,O) Rheological properties of control hydrogel (N) and drug‐loaded (O) hydrogel. In vitro experiments: n = 3. Statistical methods: t‐test for two groups, ANOVA‐Tukey for multiple groups. Data are presented as mean ± standard deviation (^*^ *p* < 0.05).

The hydrogel showed excellent injectability (Figure 3A), with smooth flow through an 18G needle, which is critical for intratumoral administration, and self‐healing capability (Figure 3B), rapidly recovering its structural integrity after mechanical disruption. Structural alterations of hydrogels before and after drug loading were analyzed using scanning electron microscopy (SEM) images (Figure 3C). Both the blank and drug‐loaded hydrogels displayed a porous network structure, with the pore morphology undergoing a slight change after drug loading. Prior to drug loading, the pores of the hydrogel presented a regular layered cavity structure, featuring relatively smooth edges and good network connectivity. After drug loading, the pores of the hydrogel transformed into an irregular network, exhibiting a more intricate pore morphology, and more filamentous connections emerged between the matrixes. The diameter of the large pores before drug loading was approximately 50–80 µm. Conversely, after drug loading, the pores were more dispersed and smaller in size (mostly 30–60 µm), suggesting that drug molecules filled the pores and bonded with the matrix. The SEM was carried out at a low magnification of 100× and a high magnification of 200×, with an electron gun acceleration voltage of 3.00 kV. Figure 3D illustrates the swelling and degradation behaviors of blank and drug‐loaded hydrogels at various time intervals. The drug‐loaded hydrogel maintains a relatively controlled swelling state within 72 h. Compared to the blank hydrogel, the drug‐loaded hydrogel exhibited a lower degree of swelling at 72 h (Figure 3E, *p* < 0.05), indicating a more stable drug release profile. This characteristic helps prevent potential toxic and adverse effects associated with sudden drug release, thereby facilitating sustained and controlled drug delivery and enhancing therapeutic efficacy. Both blank and drug‐loaded hydrogels were observed to degrade within 14 days, demonstrating favorable biodegradability.

To verify the pH/ROS responsiveness of the hydrogel in a simulated TME (Figure 3F), we conducted experiments under conditions mimicking the acidic and high ROS environments typically found in tumors. The left vial simulates an acidic environment, while the right vial simulates a high‐ROS condition. The hydrogel exhibited gradual swelling and degradation in pH (5) and H_2_O_2_ (1 mm) solutions, thereby enabling intelligent regulation of precise drug release at the tumor site and minimal release in normal tissues. In the single‐drug release studies of Cl‐amidine (Figure 3G) and enoblituzumab (Figure 3H), the release rate was fastest under pH 5.0 + H_2_O_2_ conditions, with approximately 70%–80% of the drug released within about 7 days. In contrast, the pH 7.4 group exhibited the slowest release, with only about 30%–40% released over 14 days. These results indicate that the hydrogel accelerates drug release in the acidic, high‐ROS TME while maintaining slower release under physiologically neutral conditions. Furthermore, the release profiles of Cl‐amidine and enoblituzumab were highly consistent, with the pH 5.0 + H_2_O_2_ group consistently demonstrating the fastest release and the pH 7.4 group remaining the slowest. This consistency suggests that the environment‐responsive drug release behavior of the hydrogel is independent of the drug type, demonstrating the universality of this drug‐loading system. Additionally, drug release under pH 5.0 was quicker than under pH 7.4 but slower than under pH 5.0 + H_2_O_2_ conditions, indicating a synergistic effect between acidity and high ROS levels within the TME. In the hydrogel, CY7 was added as a fluorescent substance, which can be excited under red light to visualize the grid‐like structure of the hydrogel (Figure 3I, white arrows, scale bars: 100 µm). Results of the dual‐drug synchronous release curves (Figure 3J–M) showed that the dual‐drug release in the pH 7.4 group was slow, with only 30%–40% released after 14 days. In contrast, in the pH 5.0 + H_2_O_2_ group, nearly 80% was released in about 7 days, suggesting that the TME promoted the synergistic release of the two drugs. Rheological analysis (Figure 3N,O) showed that the hydrogel predominantly exhibited elastic deformation. Crucially, drug loading did not affect its mechanical properties, with the material's elastic‐viscous behavior remaining stable.

In vitro drug release profiles (Figure S5A–H) demonstrated that the non‐responsive hydrogel exhibited relatively consistent cumulative release kinetics of Cl‐amidine and Enoblituzumab across all pH/H_2_O_2_ conditions, with no significant differences observed (*p* > 0.05). In contrast, the pH‐responsive hydrogel exhibited significantly enhanced release of both therapeutic agents in acidic media (pH 5.0) compared to neutral conditions (pH 7.4) (^*^ *p* < 0.05). The ROS‐responsive hydrogel showed a significant increase in the release of Cl‐amidine and Enoblituzumab under 1 mm H_2_O_2_ conditions (^*^ *p* < 0.05). Notably, the dual‐responsive hydrogel displayed the most prominent cumulative release of Cl‐amidine and Enoblituzumab in the combined stimulus environment (pH 5.0 + 1 mm H_2_O_2_), with release rates significantly higher than those under neutral or single‐stimulus conditions (^*^ *p* < 0.05). In vivo pharmacokinetic profiles (Figure S5I–L) revealed that the intratumoral dual‐drug group attained significantly higher concentrations of Cl‐amidine and Enoblituzumab in tumor tissues compared to the intravenous dual‐drug group (^*^ *p* < 0.05). Conversely, serum concentrations of Cl‐amidine and Enoblituzumab in the intratumoral administration group were significantly lower than those in the intravenous group (^*^ *p* < 0.05), indicating a marked reduction in systemic exposure in the intratumoral dual‐drug group.

### 3.3 Antitumor Efficacy of Hydrogel@Anti‐B7‐H3&Cl‐amidine in Orthotopic OSCC Model

**FIGURE 4:**
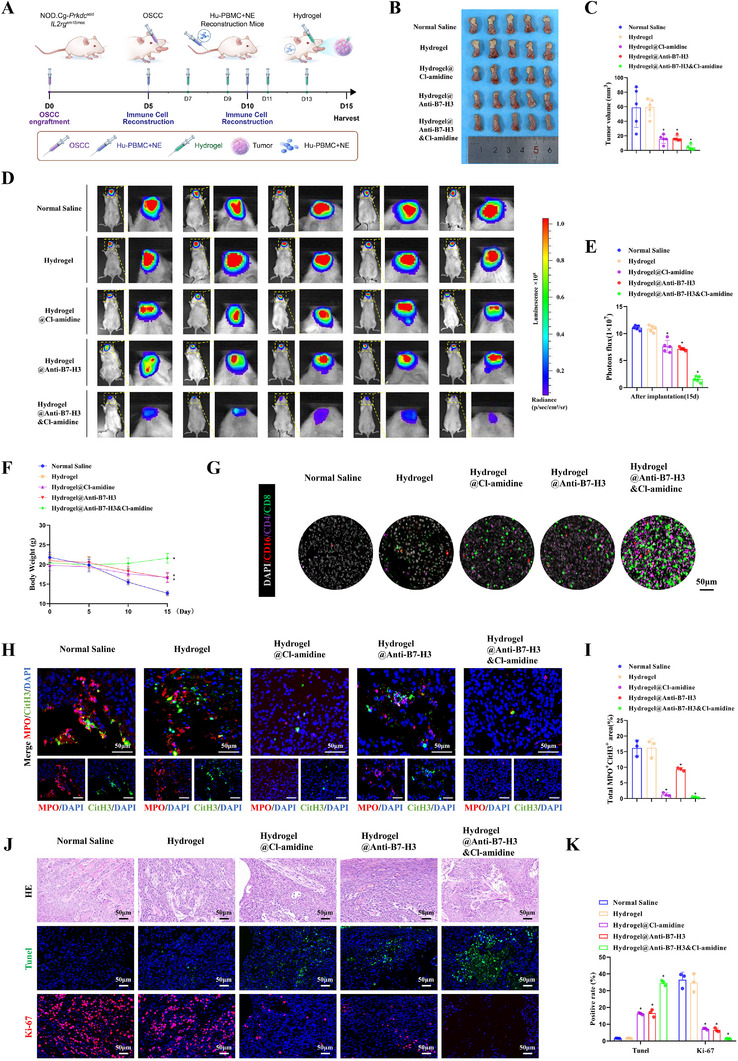
Antitumor efficacy of Hydrogel@Anti‐B7‐H3&Cl‐amidine in orthotopic OSCC animal model. (A) Experimental workflow of intratumoral hydrogel therapy. Orthotopic model via tongue submucosal injection of 1 × 10^7^ CAL‐27 cells. *NOD.Cg*‐*Prkdc* ^scid^ *Il2rg* ^em1Smoc^ mice were used as transplantation recipients to establish humanized immune system mouse models. (B) Macroscopic tumor morphology across treatment groups: saline, blank hydrogel, Anti‐B7‐H3 monotherapy hydrogel, Cl‐amidine monotherapy hydrogel, and Hydrogel@Anti‐B7‐H3&Cl‐amidine. (C) Statistical analysis of tumor volume. (D) In vivo bioluminescence imaging of orthotopic tumor‐bearing mice in each treatment group at day 15. (E) Quantitative analysis of luminescence intensity in mice using IVIS Spectrum system after specified treatments. (F) Body weight changes in mice after specified treatments. (G) IF staining of CD16 (red), CD4 (purple), and CD8 (green) in OSCC specimens from mice after orthotopic tumor treatment (scale bars: 50 µm). (H) IF staining of NETs (CitH3^+^ green, MPO^+^ red) in OSCC specimens from mice after orthotopic tumor treatment (scale bars: 50 µm). (I) Quantitative analysis of NETs density (CitH3^+^/MPO^+^ area). (J) HE staining, TUNEL, and Ki‐67 IF staining of orthotopic tumors in each treatment group (scale bars: 50 µm). (K) Statistical analysis of TUNEL and Ki‐67 IF staining. In vivo experiments: The sample size of groups A‐F was n = 5 per group, and data in groups G‐K were statistically analyzed using 3 randomly selected representative mice per group. ANOVA‐Tukey test was used for multiple‐group comparisons. Data are presented as mean ± standard deviation, and *p* < 0.05 indicates statistically significant differences.

To assess the therapeutic potential of Hydrogel@Anti‐B7‐H3&Cl‐amidine for OSCC, we established orthotopic tumor models in humanized immune system mice and performed intratumoral hydrogel administration following the workflow in Figure 4A. Donor 1‐reconstituted mice exhibited stable body weight, minimal graft‐versus‐host disease (GVHD), and significantly higher proportions of hCD3^+^ T cells and hCD66b^+^ neutrophils within the human leukocyte population compared to Donor 2 (^*^ *p* < 0.05). In contrast, Donor 3 induced severe GVHD accompanied by progressive weight loss. Therefore, Donor 1 was chosen for immune reconstitution (Figure S1K–M). Macroscopic tumor morphology of orthotopic OSCC (Figure 4B) and tumor volume measurements (Figure 4C) demonstrated that Hydrogel@Anti‐B7‐H3&Cl‐amidine significantly inhibited tumor growth compared to treatment with normal saline, blank hydrogel, or monotherapies (Anti‐B7‐H3 or Cl‐amidine alone) (^*^ *p* < 0.05). We monitored the progression of OSCC in mice from each treatment group at day 15 using small animal in vivo imaging technology. In vivo bioluminescence imaging (Figure 4D) and quantification of luminescence intensity (Figure 4E) further confirmed that tumor progression was significantly delayed in the Hydrogel@Anti‐B7‐H3&Cl‐amidine group compared to control groups (normal saline, blank hydrogel, or monotherapies), with lower luminescence intensity observed (^*^ *p* < 0.05). Monitoring of body weight changes (Figure 4F) revealed that mice in the saline and blank hydrogel groups exhibited significant weight loss, while those in the Hydrogel@Anti‐B7‐H3&Cl‐amidine treatment group maintained stable body weight and even showed an increase by day 15 (^*^ *p* < 0.05). These results indicate that the Hydrogel@Anti‐B7‐H3&Cl‐amidine effectively suppressed OSCC growth while maintaining good systemic tolerance.

IF staining (Figure 4G) and quantitative analysis (Figure S1H) demonstrated increased infiltration of CD8^+^ T cells (green), CD4^+^ T cells (purple), and CD16^+^ immune cells (red) in the Hydrogel@Anti‐B7‐H3&Cl‐amidine group compared to normal saline, blank hydrogel, or monotherapy controls (^*^ *p* < 0.05). These results indicate that the Hydrogel@Anti‐B7‐H3&Cl‐amidine effectively reprogrammed the immunosuppressive TME into an immunologically hot state. This enhanced immune infiltration reflects the synergistic effect of Anti‐B7‐H3 (activating T cells) and Cl‐amidine (reducing NETs formation), which together transform the TME from cold to hot. To evaluate the inhibition of NETs, IF staining for CitH3^+^ (green) and MPO^+^ (red) was performed (Figure 4H), with corresponding quantitative analysis (Figure 4I) revealing a significant reduction in NETs density in the Hydrogel@Anti‐B7‐H3&Cl‐amidine group versus controls (^*^ *p* < 0.05). This confirms Cl‐amidine's efficacy in suppressing NETs formation. TUNEL and Ki‐67 IF staining (Figure 4J, representative images; Figure 4K, quantitative analysis) demonstrated a significant increase in TUNEL^+^ apoptotic cells and a corresponding decrease in Ki‐67^+^ proliferating cells in the Hydrogel@Anti‐B7‐H3&Cl‐amidine group compared to the saline, blank hydrogel, and monotherapy control groups (Anti‐B7‐H3 or Cl‐amidine alone) (^*^ *p* < 0.05). These alterations are directly associated with the reduced tumor volume observed in Figure 4B, thereby confirming the synergistic effect of Hydrogel@Anti‐B7‐H3&Cl‐amidine in promoting apoptosis and inhibiting cell proliferation.

In orthotopic OSCC mice, the Hydrogel@Anti‐B7‐H3&Cl‐amidine group exhibited the smallest tumor volume and best therapeutic efficacy (Figure S6A,B, ^*^ *p* < 0.05). Normal Saline and the Free‐Anti‐B7‐H3&Cl‐amidine group exhibited significant continuous weight loss, while the Hydrogel@Anti‐B7‐H3&Cl‐amidine group maintained stable weight (Figure S6C, ^*^ *p* < 0.05). Compared with the Free‐Anti‐B7‐H3&Cl‐amidine group, the Hydrogel@Anti‐B7‐H3&Cl‐amidine group had the lowest Ki67‐positive rate and the highest TUNEL‐positive rate (Figure S6D–F, ^*^ *p* < 0.05). For biosafety, the Free‐Anti‐B7‐H3&Cl‐amidine group induced liver injury (Figure S6G), with multiple necrotic foci, inflammatory cell infiltration, partial hepatocellular degeneration, and disappearance of the structure of some hepatic lobules observed in liver tissues, along with mild ALT/T‐BIL elevation (Figure S6H,I, ^*^ *p* < 0.05).

In vivo bioluminescence imaging (Figure S7A,B) showed no significant difference in tumor burden between Blank and Isotype Control‐loaded Hydrogel groups (*p* > 0.05), while the Anti‐B7‐H3 Hydrogel group had notably reduced tumor volume (^*^ *p* < 0.05). Body weight monitoring (Figure S7C) indicated stable weight in the Anti‐B7‐H3 Hydrogel group during treatment. The two control groups exhibited high Ki‐67 expression, minimal TUNEL‐positive apoptotic cells, and no intergroup differences (*p* > 0.05), whereas the Anti‐B7‐H3 Hydrogel group showed downregulated Ki‐67 and increased apoptosis (Figure S7D–F, ^*^ *p* < 0.05). For T cell infiltration (Figure S7G–I), controls had low CD4^+^/CD8^+^ T cell counts, while the Anti‐B7‐H3 Hydrogel group displayed enhanced infiltration of both subsets (^*^ *p* < 0.05). Additionally, the Anti‐B7‐H3 Hydrogel group had significantly higher TNF‐α, IFN‐γ, Perforin, and Granzyme B expression than controls (Figure S7J–L, ^*^ *p* < 0.05). In summary, Hydrogel@Anti‐B7‐H3&Cl‐amidine effectively suppressed OSCC progression in orthotopic tumor models through modulation and reprogramming of the tumor immune microenvironment.

### 3.4 Antitumor Efficacy of Hydrogel@Anti‐B7‐H3&Cl‐amidine in Subcutaneous OSCC Model

**FIGURE 5:**
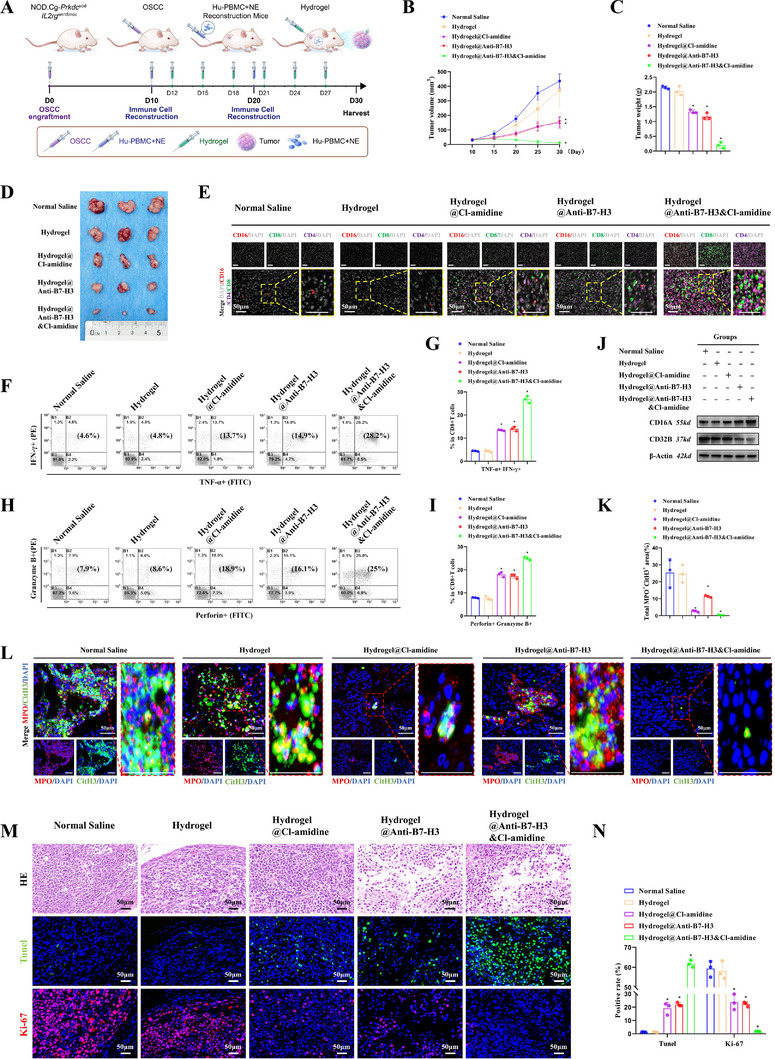
Antitumor efficacy of Hydrogel@Anti‐B7‐H3&Cl‐amidine in subcutaneous OSCC animal model. (A) Experimental workflow of intratumoral hydrogel therapy using humanized immune system mouse models. Subcutaneous model via dorsal injection of 1 × 10^7^ CAL‐27 cells. (B) Statistical analysis of tumor volume across treatment groups: saline, blank hydrogel, single‐drug Anti‐B7‐H3‐loaded hydrogel, single‐drug Cl‐amidine‐loaded hydrogel, and Hydrogel@Anti‐B7‐H3&Cl‐amidine. (C) Statistical analysis of tumor weight in each treatment group. (D) Antitumor efficacy at day 30 post‐treatment. (E) IF staining of CD16 (red), CD4 (purple), and CD8 (green) in subcutaneous tumor specimens (scale bars: 50 µm). (F) Representative flow cytometry plots showing TNF‐α and IFN‐γ expression in tumor‐infiltrating CD8^+^ T cells. (G) Statistical analysis of flow cytometry results for CD8^+^ T cells. (H) Representative flow cytometry plots showing perforin and granzyme B expression in tumor‐infiltrating CD8^+^ T cells. (I) Statistical analysis of flow cytometry results for CD8^+^ T cells. (J) Protein expression of CD16A and CD32B in subcutaneous tumors. (K, L) IF staining of NETs (CitH3^+^ green, MPO^+^ red) in subcutaneous tumors (scale bars: 50 µm) and quantitative analysis of IF intensity (Total MPO^+^ CitH3^+^ area). (M) HE staining, TUNEL (green), and Ki‐67 (red) IF staining of subcutaneous tumors (scale bars: 50 µm). (N) Statistical analysis of TUNEL and Ki‐67 IF staining. In vivo experiments: n = 3 per group. Statistical methods: ANOVA‐Tukey test for multiple‐group comparisons. Data are presented as mean ± SD, and *p* < 0.05 indicates statistically significant differences.

To further validate the therapeutic universality of Hydrogel@Anti‐B7‐H3&Cl‐amidine across OSCC models, we established subcutaneous OSCC xenografts in mice with a humanized immune system and administered intratumoral injections of the hydrogel according to the workflow illustrated in Figure 5A. Statistical analysis of tumor volume (Figure 5B) and weight (Figure 5C) demonstrated that Hydrogel@Anti‐B7‐H3&Cl‐amidine significantly suppressed subcutaneous tumor growth compared to normal saline, blank hydrogel, or monotherapy groups (^*^ *p* < 0.05). By day 30, the Hydrogel@Anti‐B7‐H3&Cl‐amidine group exhibited the smallest tumor volume and weight (Figure 5D). These results confirmed the consistent antitumor efficacy of the Hydrogel@Anti‐B7‐H3&Cl‐amidine across both orthotopic and subcutaneous OSCC models.

IF staining of subcutaneous tumor sections (Figure 5E) and quantitative analysis (Figure S1J) revealed increased infiltration of CD8^+^ (green), CD4^+^ (purple) T cells, and CD16^+^ (red) immune cells in the Hydrogel@Anti‐B7‐H3&Cl‐amidine group compared to normal saline, blank hydrogel, or monotherapy controls (^*^ *p* < 0.05). Flow cytometry further indicates potential functional enhancement of CD8^+^ T cells in the Hydrogel@Anti‐B7‐H3&Cl‐amidine treatment group, which showed elevated expression of TNF‐α and IFN‐γ (Figure 5F,G) alongside increased levels of perforin and granzyme B (Figure 5H,I) compared to normal saline, blank hydrogel, or monotherapy controls (^*^ *p* < 0.05). These elevated cytokine and cytotoxic molecule levels are consistent with enhanced function of CD8^+^ T cells, potentially contributing to reduced tumor burden. Additionally, protein expression analysis of subcutaneous OSCC tumors (Figure 5J) and orthotopic tongue tumors (Figure S1I) revealed consistent patterns. Compared to controls, CD16A (a key ADCC receptor) was upregulated in both Hydrogel@Anti‐B7‐H3 and Hydrogel@Anti‐B7‐H3&Cl‐amidine groups, with peak expression observed in the Hydrogel@Anti‐B7‐H3&Cl‐amidine group. Conversely, CD32B (an inhibitory receptor) was significantly downregulated in all antibody‐containing groups, reaching its lowest level in the Hydrogel@Anti‐B7‐H3&Cl‐amidine group. These findings demonstrate that the Hydrogel@Anti‐B7‐H3&Cl‐amidine enhances ADCC through coordinated CD16A maximization and CD32B suppression, with consistent efficacy across both subcutaneous and orthotopic OSCC models. Consistent with orthotopic results, IF staining of subcutaneous tumors (Figure 5K,L) showed a significant reduction in NETs density (CitH3^+^/MPO^+^ positive area) in the Hydrogel@Cl‐amidine and Hydrogel@Anti‐B7‐H3&Cl‐amidine group, confirming Cl‐amidine mediated suppression of NETs formation across model systems (^*^ *p* < 0.05). TUNEL and Ki‐67 staining (Figure 5M,N) further revealed increased TUNEL^+^ apoptotic cells and decreased Ki‐67^+^ proliferating cells in the Hydrogel@Anti‐B7‐H3&Cl‐amidine group (^*^ *p* < 0.05). The single‐drug hydrogel groups exhibited significantly lower expression of the four inhibitory checkpoints (PD‐1, CTLA‐4, TIM‐3, and LAG‐3) compared to the Hydrogel and Normal Saline groups (^*^ *p* < 0.05). The Free Anti‐B7‐H3&Cl‐amidine group showed reduced checkpoint expression relative to the single‐drug hydrogel groups and control groups (^*^ *p* < 0.05). Notably, the Hydrogel@Anti‐B7‐H3&Cl‐amidine group displayed the lowest positive rates of PD‐1, CTLA‐4, TIM‐3, and LAG‐3 among all treatment groups (^*^ *p* < 0.05), indicating that immune escape was alleviated (Figure S8). These changes directly contributed to reduced tumor burden, reinforcing the synergistic effect of dual targeting B7‐H3 and NETs. In summary, Hydrogel@Anti‐B7‐H3&Cl‐amidine exhibits robust and consistent antitumor activity in subcutaneous OSCC models, driven by synergistic immune microenvironment reprogramming through B7‐H3 blockade and NETs inhibition.

### 3.5 B7‐H3 Drives OSCC Invasion and Metastasis via EMT Induction

**FIGURE 6:**
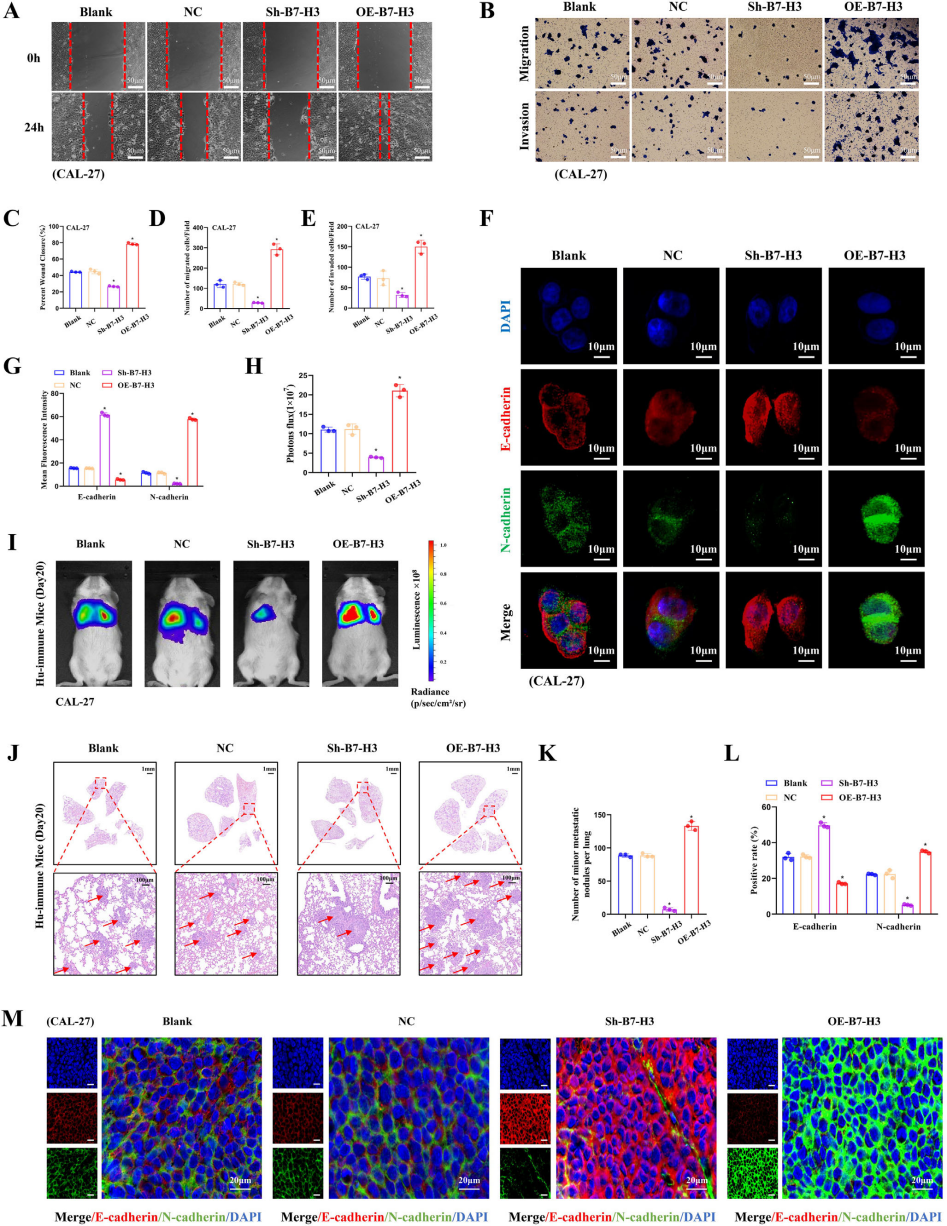
B7‐H3 promotes invasion and metastasis of OSCC via EMT activation. (A) Wound healing assay with images captured at 0 and 24 h (scale bars: 50 µm). (B) Transwell migration and invasion assay using 8 µm pore size membranes (scale bars: 50 µm). (C) Quantitative analysis of wound healing assay. (D) Quantitative analysis of Transwell migration assay. (E) Quantitative analysis of Transwell invasion assay. (F) IF staining of E‐cadherin (red) and N‐cadherin (green) in B7‐H3 knockdown and overexpressed CAL‐27 cell line. (scale bars: 10 µm). (G) Quantitative analysis of IF staining for E‐cadherin and N‐cadherin. (H) Quantitative analysis of lung fluorescence intensity at day 20 in a mouse tail vein injection lung metastasis model using CAL‐27 cells with B7‐H3 knockdown and overexpression. (I) In vivo bioluminescence imaging of lung metastases using an IVIS Spectrum system. (J) HE staining of metastatic lungs (red arrows: tumor foci) (scale bars: 1 mm for low magnification/100 µm for high magnification). (K) Quantitative analysis of lung metastatic foci. (L) Quantitative analysis of IF staining for E‐cadherin and N‐cadherin in metastatic tumors of each treatment group. (M) IF staining of E‐cadherin (red), N‐cadherin (green), and DAPI (blue) in metastatic tumors of each treatment group (scale bars: 20 µm). CAL‐27 cells were transfected with OE‐B7‐H3/Sh‐B7‐H3 plasmids to modulate B7‐H3 expression. In vitro experiments (A–G): n = 3. In vivo experiments (H–M): n = 3 per group. ANOVA‐Tukey test for multiple‐group comparisons. Data are presented as mean ± standard deviation (^*^ *p* < 0.05).

To elucidate B7‐H3's role in OSCC progression, we assessed its effects on invasion, migration, and EMT via in vitro assays, with in vivo validation of metastatic potential. Wound healing assays (Figure 6A) and quantitative analysis (Figure 6C) revealed that B7‐H3 overexpression in CAL‐27 cells significantly accelerated wound closure at 24 h post‐wounding, while B7‐H3 knockdown (Sh‐B7‐H3) delayed closure compared to controls (^*^ *p* < 0.05). Transwell assays (Figure 6B) and quantitative analysis (Figure 6D,E) revealed that B7‐H3 overexpression in CAL‐27 cells significantly increased migrated and invaded cell numbers, whereas its knockdown reduced these metrics compared to controls (^*^ *p* < 0.05). These results indicate that B7‐H3 boosts the migratory and invasive capabilities of OSCC cells in vitro. To elucidate B7‐H3's role in EMT regulation, we analyzed EMT markers via IF in CAL‐27 cells with B7‐H3 overexpression or knockdown (Figure 6F). Quantitative IF imaging (Figure 6G) demonstrated that B7‐H3 overexpression concurrently suppressed E‐cadherin (red) and elevated N‐cadherin (green), while knockdown reciprocally upregulated E‐cadherin and downregulated N‐cadherin (^*^ *p* < 0.05). Correlative analyses further showed a significant positive correlation of B7‐H3 expression levels with EMT markers (Figure S1F, correlation coefficient = 0.51, *p* < 0.001). These data indicate that B7‐H3 promotes the activation of EMT in OSCC cells. This shift in EMT markers‐characterized by loss of epithelial adhesion (E‐cadherin) and gain of mesenchymal motility (N‐cadherin)‐directly contributes to the enhanced migratory and invasive capacities observed in B7‐H3‐overexpressing cells. Compared with the OE‐B7‐H3 group, the OE‐B7‐H3+siB7‐H3 and OE‐B7‐H3+LY294002 groups showed significantly reduced wound closure rate, decreased number of migrated and invaded cells, restored E‐cadherin fluorescence intensity, and reduced N‐cadherin fluorescence intensity (^*^ *p* < 0.05) (Figure S9). This finding preliminarily supports that B7‐H3 promotes OSCC invasion, migration, and EMT in a PI3K pathway‐dependent manner.

To evaluate B7‐H3's impact on metastatic potential in vivo, bioluminescence imaging of lung metastases revealed markedly stronger fluorescence signals in mice injected with B7‐H3‐overexpressing CAL‐27 cells versus controls (Figure 6I). Quantitative analysis (Figure 6H) confirmed this effect, showing increased lung fluorescence intensity in the overexpression group and reciprocally decreased intensity following B7‐H3 knockdown (^*^ *p* < 0.05). Histopathological assessment of lung tissues (HE staining, Figure 6J) demonstrated elevated metastatic foci (red arrows) in the B7‐H3‐overexpression group versus controls, with reduced foci in knockdown specimens. Quantitative analysis (Figure 6K) confirmed this reciprocal pattern with increased foci counts after overexpression and decreased counts following knockdown (^*^ *p* < 0.05). Furthermore, IF of metastatic tissues (Figure 6L,M) recapitulated in vitro EMT patterns, showing reciprocal E‐cadherin downregulation and N‐cadherin upregulation in B7‐H3‐overexpressing tumors, whereas the Sh‐B7‐H3 group displayed the reverse expression profile (^*^ *p* < 0.05). In conclusion, collective results consistently show that B7‐H3 facilitates OSCC invasion and metastasis through EMT activation.

### 3.6 NETs Promote Invasion and Metastasis of OSCC via EMT Activation

**FIGURE 7:**
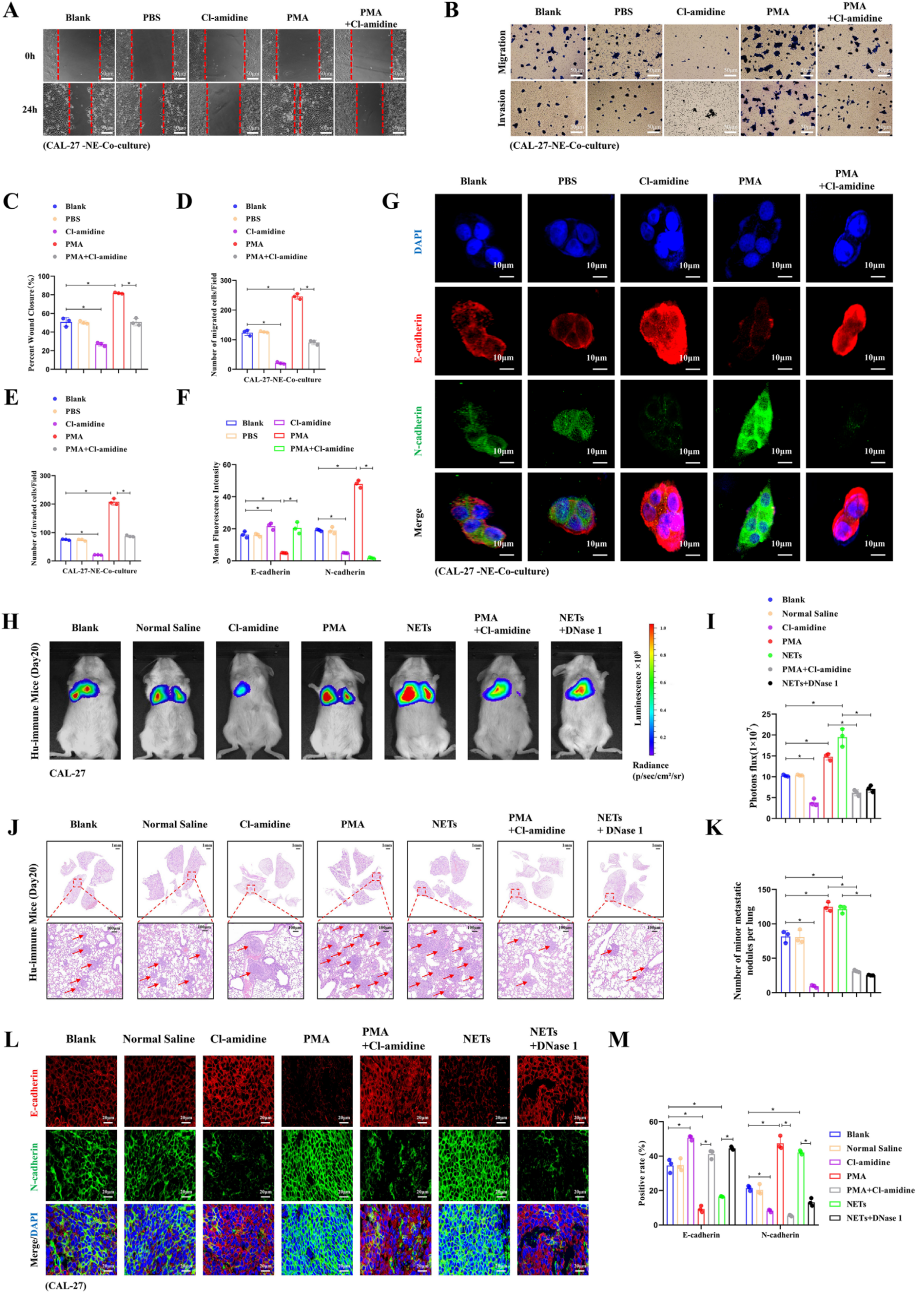
NETs promote invasion and metastasis of OSCC via EMT activation. (A) Wound healing assay of CAL‐27 cells co‐cultured with neutrophils (NE) in a transwell system (CAL‐27 in lower chamber, NE in upper chamber) across treatment groups (Blank, PBS, Cl‐amidine, PMA, and PMA + Cl‐amidine) (scale bars: 50 µm). (B) Transwell migration and invasion assay with CAL‐27 cells co‐cultured with NE (CAL‐27 in upper chamber, NE in lower chamber) (scale bars: 50 µm). Transwell inserts pre‐coated with Matrigel for invasion assay. (C) Quantitative analysis of wound healing assay. (D) Quantitative analysis of Transwell migration assay. (E) Quantitative analysis of Transwell invasion assay. (F) Quantitative analysis of IF staining for E‐cadherin and N‐cadherin. (G) IF staining of E‐cadherin (red) and N‐cadherin (green) in CAL‐27 cells co‐cultured with NE under different treatments (scale bars: 10 µm). (H) In vivo bioluminescence imaging of lung metastases in a mouse tail vein injection lung metastasis model (injected with CAL‐27 cells) at day 20 post‐treatment. (I) Quantitative analysis of fluorescence intensity in metastatic tumors. (J) HE staining of metastatic lungs, with red arrows indicating metastatic foci (scale bars: 1 mm for low magnification/100 µm for high magnification). (K) Quantitative analysis of lung metastatic foci. (L) IF staining of E‐cadherin (red), N‐cadherin (green), and DAPI (blue) in metastatic tumors of each treatment group (scale bars: 20 µm). (M) Quantitative analysis of IF staining for E‐cadherin and N‐cadherin in metastatic tumors. In vitro experiments (A–G): n = 3. In vivo experiments (H–M): n = 3 per group. ANOVA‐Tukey test for multiple‐group comparisons. Data are presented as mean ± standard deviation (^*^ *p* < 0.05).

To clarify NETs' role in OSCC progression, we assessed their effects on invasion, migration, and EMT via co‐culture assays, with in vivo validation of metastatic potential. Wound healing assays were conducted using a transwell co‐culture system across five experimental groups: Blank, PBS, Cl‐amidine, PMA, and PMA + Cl‐amidine (Figure 7A). Quantitative analysis (Figure 7C) indicated that, compared to the blank or PBS controls, co‐culture with PMA‐treated neutrophils significantly accelerated wound closure in CAL‐27 cells at 24 h post‐wounding (^*^ *p* < 0.05). In contrast, treatment with Cl‐amidine resulted in delayed wound closure relative to the control groups (^*^ *p* < 0.05). Importantly, when compared to the PMA‐treated group, the PMA + Cl‐amidine group showed a significant reversal of PMA‐induced acceleration (^*^ *p* < 0.05). Transwell migration and invasion assays (Figure 7B), along with corresponding quantitative analyses demonstrated that induction of NETs via PMA‐treated neutrophils markedly increased the number of migrated (Figure 7D) and invaded (Figure 7E) CAL‐27 cells (^*^ *p* < 0.05). Conversely, inhibition of NETs via Cl‐amidine led to reduced migration and invasion relative to control groups (^*^ *p* < 0.05). Importantly, versus the PMA group, the PMA + Cl‐amidine group significantly reversed both PMA‐induced migration promotion and invasiveness enhancement (^*^ *p* < 0.05). These findings collectively suggest that NETs enhance OSCC cells' migratory and invasive abilities in vitro. Correlative analyses revealed a significantly positive association between MPO expression and EMT marker levels (Figure S1G, correlation coefficient = 0.284, *p* = 8.16e‐11). To investigate NETs' role in EMT regulation, we analyzed EMT markers by IF in CAL‐27 cells co‐cultured with NE under the above treatments (Figure 7G). Quantitative IF imaging (Figure 7F) revealed that the PMA group suppressed E‐cadherin while increasing N‐cadherin (^*^ *p* < 0.05). In contrast, the Cl‐amidine group upregulated E‐cadherin and downregulated N‐cadherin (^*^ *p* < 0.05). Importantly, versus the PMA group, the PMA + Cl‐amidine group significantly reversed EMT marker changes (^*^ *p* < 0.05). This shift in EMT marker expression directly contributes to the increased migratory and invasive potential observed in NETs‐exposed OSCC cells.

To evaluate NETs' impact on in vivo metastatic potential, we established a lung metastasis model in humanized immune mice via tail vein injection of CAL‐27 cells, with treatment groups including blank, normal saline, Cl‐amidine, PMA, NETs, PMA + Cl‐amidine, and NETs + DNase 1. Bioluminescence imaging at day 20 post‐treatment (Figure 7H) and quantitative analysis (Figure 7I) revealed that, compared to control groups, both the PMA‐treated and NETs‐treated groups showed significantly stronger lung fluorescence signals, indicating enhanced metastatic burden (^*^ *p* < 0.05). In contrast, the Cl‐amidine group exhibited markedly weaker lung fluorescence signals relative to controls, suggesting reduced metastatic potential (^*^ *p* < 0.05). The PMA + Cl‐amidine group showed significantly attenuated lung fluorescence compared to the PMA group, demonstrating that Cl‐amidine reversed PMA's pro‐metastatic effect. Similarly, the NETs + DNase 1 group displayed weakened fluorescence relative to the NETs group, confirming that DNase 1 reversed NETs' pro‐metastatic effect (^*^ *p* < 0.05). Histopathological evaluation of lung tissues via HE staining (Figure 7J) and quantitative analysis (Figure 7K) revealed that, compared to control groups, the PMA‐treated and NETs‐treated groups exhibited a significantly increased number of metastatic foci (red arrows), indicating enhanced metastatic burden (^*^ *p* < 0.05). Conversely, the Cl‐amidine group exhibited a marked reduction in metastatic foci relative to controls, suggesting reduced metastatic potential (^*^ *p* < 0.05). Notably, the PMA + Cl‐amidine group displayed significantly fewer metastatic foci compared to the PMA group, demonstrating that Cl‐amidine reversed PMA's pro‐metastatic effect. Similarly, the NETs + DNase 1 group exhibited fewer foci relative to the NETs group, confirming that DNase 1 reversed NETs' pro‐metastatic effect (^*^ *p* < 0.05). Moreover, IF staining of lung metastatic tumor specimens (Figure 7L) and quantitative analysis (Figure 7M) revealed EMT marker changes consistent with in vivo metastatic trends: compared to control groups, the PMA and NETs groups exhibited downregulated E‐cad and upregulated N‐cad, indicating EMT activation (^*^ *p* < 0.05). Conversely, the Cl‐amidine group displayed the opposite trend relative to controls (^*^ *p* < 0.05). Notably, the Cl‐amidine reversed the EMT marker changes induced by PMA. Similarly, the DNase 1 reversed the NETs‐induced EMT (^*^ *p* < 0.05). In conclusion, collective experimental data consistently show that NETs facilitate OSCC invasion and metastasis via EMT activation.

### 3.7 Validation of B7‐H3 and NETs Promoting OSCC Metastasis via the EMT Pathway in the SCC‐9 Cell Line

To exclude cell line specificity and strengthen the generalizability of our findings, we validated the roles of B7‐H3 and NETs in regulating EMT and OSCC progression using the SCC9 cell line. For B7‐H3, in vitro assays (Figure S2A–G) showed consistent trends with CAL‐27 cells, as B7‐H3 overexpression in SCC9 cells accelerated wound closure, increased migration and invasion, and induced EMT marker shifts, including downregulated E‐cadherin and upregulated N‐cadherin, while B7‐H3 knockdown reversed these effects (^*^ *p* < 0.05). For NETs, co‐culture experiments (Figure S2H–N) revealed that PMA‐induced NETs promoted SCC9 cell migration, invasion, and EMT activation characterized by suppressed E‐cadherin and elevated N‐cadherin, whereas the Cl‐amidine‐treated group inhibited these effects (^*^ *p* < 0.05). Notably, the PMA + Cl‐amidine group reversed PMA‐induced changes (^*^ *p* < 0.05), mirroring CAL‐27 data.

We developed a lung metastasis model in humanized immune mice via tail vein injection of B7‐H3‐overexpressing SCC‐9 cells, with treatment groups including blank, normal saline, Cl‐amidine, anti‐B7‐H3, and anti‐B7‐H3+Cl‐amidine. Bioluminescence imaging (Figure S2O,R) revealed that the B7‐H3‐overexpressing group exhibited significantly stronger lung fluorescence signals, indicating enhanced metastatic burden, whereas the Cl‐amidine, anti‐B7‐H3, and anti‐B7‐H3+Cl‐amidine groups showed weaker signals (^*^ *p* < 0.05). Notably, the anti‐B7‐H3+Cl‐amidine group displayed the weakest fluorescence, reflecting the most potent inhibitory effect on metastasis (^*^ *p* < 0.05). HE staining of lung tissues (Figure S2P,S) confirmed these trends: compared to control groups, the Cl‐amidine, anti‐B7‐H3, and anti‐B7‐H3+Cl‐amidine groups showed a marked reduction in metastatic foci, with the anti‐B7‐H3+Cl‐amidine group exhibiting the fewest foci (^*^ *p* < 0.05). IF staining of metastatic tumors (Figure S2Q,T) recapitulated EMT marker changes: B7‐H3 overexpression reduced E‐cadherin and elevated N‐cadherin, while the anti‐B7‐H3+Cl‐amidine group significantly reversed this pattern (^*^ *p* < 0.05). These results validate that B7‐H3 and NETs promote metastasis in SCC‐9 cells via EMT. Importantly, combined intervention targeting B7‐H3 and NETs exerts the most significant inhibitory effect on tumor metastasis.

### 3.8 In Vivo Safety Evaluation of Hydrogel@Anti‐B7‐H3&Cl‐amidine

To evaluate the safety profile of Hydrogel@Anti‐B7‐H3&Cl‐amidine, five treatment groups were established in healthy mice: normal saline, Hydrogel, Hydrogel@Cl‐amidine, Hydrogel@Anti‐B7‐H3, and Hydrogel@Anti‐B7‐H3&Cl‐amidine. Histopathological analysis using H&E staining of major organs (Figure S3A) exhibited no notable pathological alterations, such as inflammation, necrosis, or structural damage, across any of the treatment groups. The tissue morphology in all groups, including those administered with Hydrogel, Hydrogel@Cl‐amidine, Hydrogel@Anti‐B7‐H3, and Hydrogel@Anti‐B7‐H3&Cl‐amidine, was comparable to that observed in the control group, with no visible signs of organ injury (scale bars: 100 µm). Additionally, blood biochemical assessments (Figure S3B–P) corroborated these findings. Key markers of hepatic function (ALT, AST, DBIL, T‐BIL, ALB, ALP, γ‐GT, TBA), renal function (BUN, UREA, CREA, UA), and cardiac/muscle function (CK, LDH, LDH1) displayed no statistically significant differences between the treatment groups (*p* > 0.05). Taken together, the Hydrogel@Anti‐B7‐H3&Cl‐amidine exhibits excellent in vivo biocompatibility without inducing apparent toxicity to major organs or impairing physiological functions.

## 4 Discussion

OSCC remains a challenging malignancy with limited efficacy from current immunotherapies, emphasizing the critical need for novel strategies targeting immune escape mechanisms and metastatic progression [33]. This study addresses this gap by elucidating the synergistic role of B7‐H3 and NETs in OSCC pathogenesis and introducing a dual‐targeting, pH/ROS‐responsive hydrogel system that addresses key limitations of conventional therapeutic approaches. A significant finding is the coordinated immunosuppressive interaction between B7‐H3 and NETs within the OSCC TME. Analysis of clinical specimens demonstrated that B7‐H3 overexpression correlates with increased NETs accumulation, both inversely associated with T cell (CD4^+^, CD8^+^) infiltration. Our previous research suggested that OSCC cells expressing B7‐H3 mediate immune escape by inhibiting CD8^+^ T cell activity [10]. In vitro experiments suggested that NETs impair CD8^+^ T cell function by reducing the secretion of cytotoxic molecules, thereby complementing the known inhibitory effects of B7‐H3 on T cell activity. Building on these findings, we investigated the interaction between B7‐H3 and NETs. Figure S4 demonstrates a direct regulatory relationship, in which B7‐H3 overexpression in OSCC cells drives NETs formation in neutrophils. Together, these observations highlight the potential therapeutic value of simultaneously targeting B7‐H3 and NETs. By co‐targeting these pathways, our hydrogel system effectively reprograms the immunosuppressive TME. These findings are supported by the observed upregulation of CD16A and downregulation of CD32B in treated tumors, which collectively enhance antibody‐dependent cellular cytotoxicity (ADCC) and reverse the immune desert phenotype. Isotype control experiments confirm that the antitumor efficacy of the hydrogel system is specifically mediated by Enoblituzumab targeting B7‐H3. Figure S8 further confirms that Hydrogel@Anti‐B7‐H3&Cl‐amidine can efficaciously downregulate the expression of key inhibitory checkpoints (PD‐1, CTLA‐4, TIM‐3, and LAG‐3) in the tumor immune microenvironment, thereby achieving genuine alleviation of immune escape.

Mechanistically, B7‐H3 and NETs promote OSCC metastasis by activating the EMT pathway. In vitro studies showed that B7‐H3 overexpression or NETs formation caused EMT marker changes, specifically lower E‐cad and higher N‐cad, enhancing cell motility and invasiveness. Conversely, inhibition of these factors reversed the EMT process. In NSCLC, EMT is promoted by B7‐H3 through the PI3K/AKT‐SIRT1 axis [34], whereas NETs trigger EMT and enhance metastasis by inhibiting lncRNA MIR503HG [35]. This cross‐cancer evidence underscores the universal role of B7‐H3 and NETs in facilitating EMT during tumor development. Notably, rescue experiments further support that B7‐H3 promotes OSCC invasion, migration, and EMT in a PI3K pathway‐dependent manner. In vivo, anti‐B7‐H3 treatment, Cl‐amidine administration, or their combination significantly reduced lung metastatic burden, with the combined regimen showing the greatest efficacy. Cross‐validation using SCC‐9 cells confirmed that this EMT‐driven metastatic mechanism is not restricted to a specific cell line, suggesting broader applicability. Clinically, the positive correlation between B7‐H3/NETs levels and EMT markers in patient samples emphasizes the link between immune evasion and metastatic progression, reinforcing the therapeutic potential of co‐targeting these pathways to address both local tumor growth and distant metastasis.

The pH/ROS‐responsive hydrogel [36, 37], unlike systemic antibody administration, enables targeted therapeutic release within the acidic, high‐ROS tumor microenvironment [38]. Compared with the direct local injection of free dual drugs, the pH/ROS‐responsive hydrogel co‐delivery system not only enhanced antitumor efficacy but also exhibited superior biosafety. This precision minimizes systemic exposure, as demonstrated by normal liver, kidney, and cardiac function markers in safety assessments, and avoids the off‐target effects commonly seen with intravenous therapies. The hydrogel's injectability and self‐healing properties facilitate minimally invasive intratumoral administration [39]. Importantly, the dual‐stimulus responsiveness to both pH and ROS ensures reliable drug release across heterogeneous tumor environments. This design contributed to the hydrogel's superior antitumor efficacy compared to monotherapies, with the combination group demonstrating the smallest tumor volumes, lowest metastatic burden, and highest T cell infiltration across experimental models. Additionally, silver nanoparticle mucoadhesive hydrogel with PD‐1 antibody in mouse OSCC models modulates oral microbiota, offering a novel local OSCC therapy [40].

These findings address significant limitations of current B7‐H3‐targeted therapies. The clinical limitations of enoblituzumab, including systemic toxicity, potentially stem from off‐target binding and broad biodistribution [41]. In contrast, our localized hydrogel delivery achieves high intratumoral drug concentrations, with minimal systemic leakage, as confirmed by the absence of organ pathology or biochemical abnormalities. Recent studies observed that NETs can trap CD8^+^ T cells in the tumor stroma, preventing them from infiltrating the tumor site to kill cancer cells [22, 42, 43]. Our hydrogel system, however, achieves a synergistic therapeutic enhancement through the combination of B7‐H3 blockade and NETs inhibition: anti‐B7‐H3 reinvigorates T cell activity, while Cl‐amidine degrades the NETs barrier to facilitate T cell infiltration. This synergy explains the superior efficacy of the combination regimen in suppressing tumor growth and metastasis. This study has limitations, such as using humanized immune mouse models, which, although more physiologically relevant than immunodeficient models, fail to fully mirror the complexity of the OSCC TME. Furthermore, this study did not analyze the regulatory mechanism between B7‐H3 and NETs in depth, nor did it assess the long‐term efficacy of the hydrogel. Another minor limitation is that the dose relationship between the hydrogel's intratumoral release and systemic free drug delivery was not systematically validated. This is because the two routes target distinct endpoints (local tumor shrinkage vs. metastasis inhibition) with no direct comparison. These aspects will be further explored in future studies.

In conclusion, this study demonstrates that B7‐H3 and NETs synergistically contribute to the progression of OSCC. These factors compromise anti‐tumor immunity by suppressing T cell function and physically hindering T cell infiltration into the TME. Additionally, they promote tumor metastasis through the activation of the EMT pathway. Our novel dual‐targeting hydrogel system, which is responsive to both pH and ROS, effectively inhibits these mechanisms. Preclinical evaluations revealed that the hydrogel exhibits superior antitumor efficacy compared to conventional therapies. It significantly enhances T cell infiltration into tumors, restores their cytotoxic activity, and reduces metastatic spread. Through localized drug delivery, the hydrogel minimizes systemic toxicity, thereby addressing critical limitations associated with current systemic B7‐H3‐targeted treatments. Although additional research is needed, this work shows that simultaneously targeting B7‐H3 and NETs holds potential as a promising strategy to improve OSCC treatment outcomes.

## Author Contributions

H.L. designed the experiments, performed the research, and wrote the manuscript; Y.L. and Y.T.H. performed the research and analyzed the data; Y.B.Y., Y.D.H., J.L., and J.Q.T. drew the figures; Z.Y.Z., Y.W., Y.L.H., and X.L.Z. reviewed and edited the manuscript; X.J.Y. and J.H.W. acquired funding, supervised, guided, and managed the project.

## Funding

This work was supported by the National Natural Science Foundation of China (Grant Nos. 82303332, 81973114) and the Shaanxi Provincial Health Commission Research Innovation Capacity Improvement Program (Grant No. 2025PT‐11).

## Conflicts of Interest

The authors declare no conflicts of interest.

## Supporting information




**Supporting File**: advs74137‐sup‐0001‐SuppMat.docx.

## Data Availability

The data that support the findings of this study are available from the corresponding author upon reasonable request.
