# Supplementary material for: pH/ROS‐Responsive Injectable Hydrogel Co‐Loaded with B7‐H3 Blocker and NETs Suppressor Boosts OSCC Synergistic Immunotherapy
Source: Adv Sci (Weinh). 2026 Feb 13;13(20):e15431. doi: 10.1002/advs.202515431 (PMC13067762; doi:10.1002/advs.202515431)
Supplement: Supplementary file 1 — Supporting File: advs74137‐sup‐0001‐SuppMat.docx. [file ADVS-13-e15431-s001.docx]

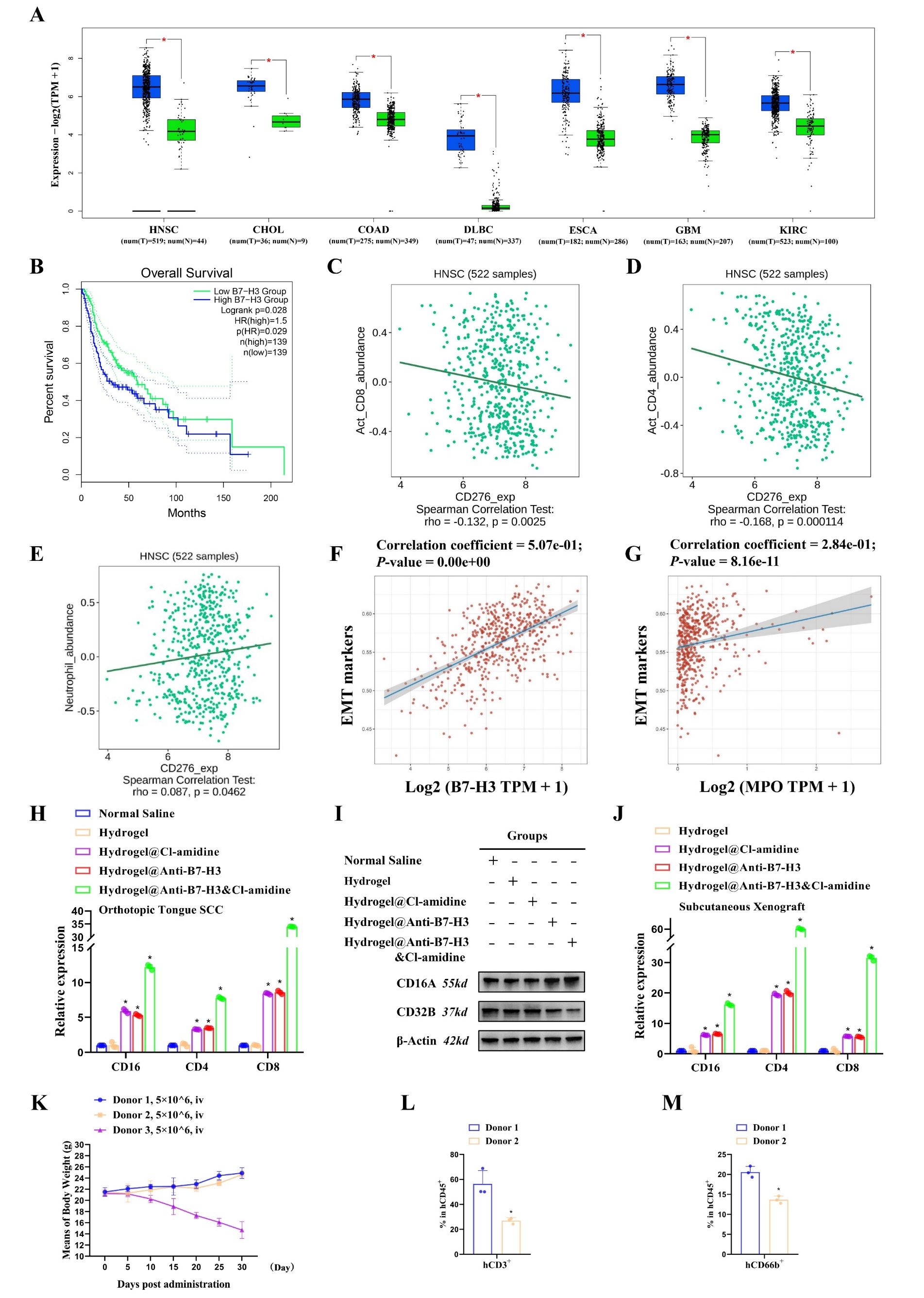


**Supplementary Figure 1. Pan-cancer expression of B7-H3 and its correlation with patient prognosis, immune cell infiltration, and EMT markers** (A) Pan-cancer expression of B7-H3 in human malignancies: HNSC (head and neck squamous cell carcinoma), CHOL (cholangiocarcinoma), COAD (colon adenocarcinoma), DLBC (lymphoid neoplasm diffuse large B-cell lymphoma), ESCA (esophageal carcinoma), GBM (glioblastoma multiforme), and KIRC (kidney renal clear cell carcinoma). (B) Association of B7-H3 expression with overall survival in HNSC patients. (C) Correlation analysis between B7-H3 expression and CD8^+^ T cell infiltration in HNSC (rho=-0.132, *p*=0.0025). (D) Correlation analysis between B7-H3 expression and CD4^+^ T cell infiltration in HNSC (rho=-0.168, *p*=0.000114). (E) Correlation analysis between B7-H3 expression and neutrophil infiltration in HNSC (rho=0.087, *p*=0.0462). (F) Correlation analysis between B7-H3 expression and EMT markers (rho=0.507, *p*<0.0001). (G) Correlation analysis between MPO expression and EMT markers (rho=0.284, *p*=8.16×10^−11^). (H) Statistical analysis of CD16, CD4, and CD8 IF staining in orthotopic tongue tumor sections of Figure 4G. (I) Protein expression of CD16A and CD32B in orthotopic tongue tumor tissues. (J) Statistical analysis of CD16, CD4, and CD8 IF staining in subcutaneous tumor specimens of Figure 5E. (K) Body weight changes in M-NSG mice following intravenous co-injection of PBMCs and neutrophils from three donors. (L) Proportion of hCD3⁺ T cells within the hCD45⁺ leukocyte population in M-NSG mice. (M) Proportion of hCD66b⁺ neutrophils within the hCD45⁺ leukocyte population in M-NSG mice. Bioinformatics analyses (A-G): Data from TCGA/ GEPIA2/ TISIDB/ TIMER2.0 databases. Spearman’s rank correlation analysis was used for correlation assays, and Kaplan-Meier survival analysis (log-rank test) was used for survival assessment. (K-M): 3 mice per donor. Statistical methods: t-test for two groups, ANOVA-Tukey test for multiple-group comparisons. Data are presented as mean ± standard deviation (**p* < 0.05).


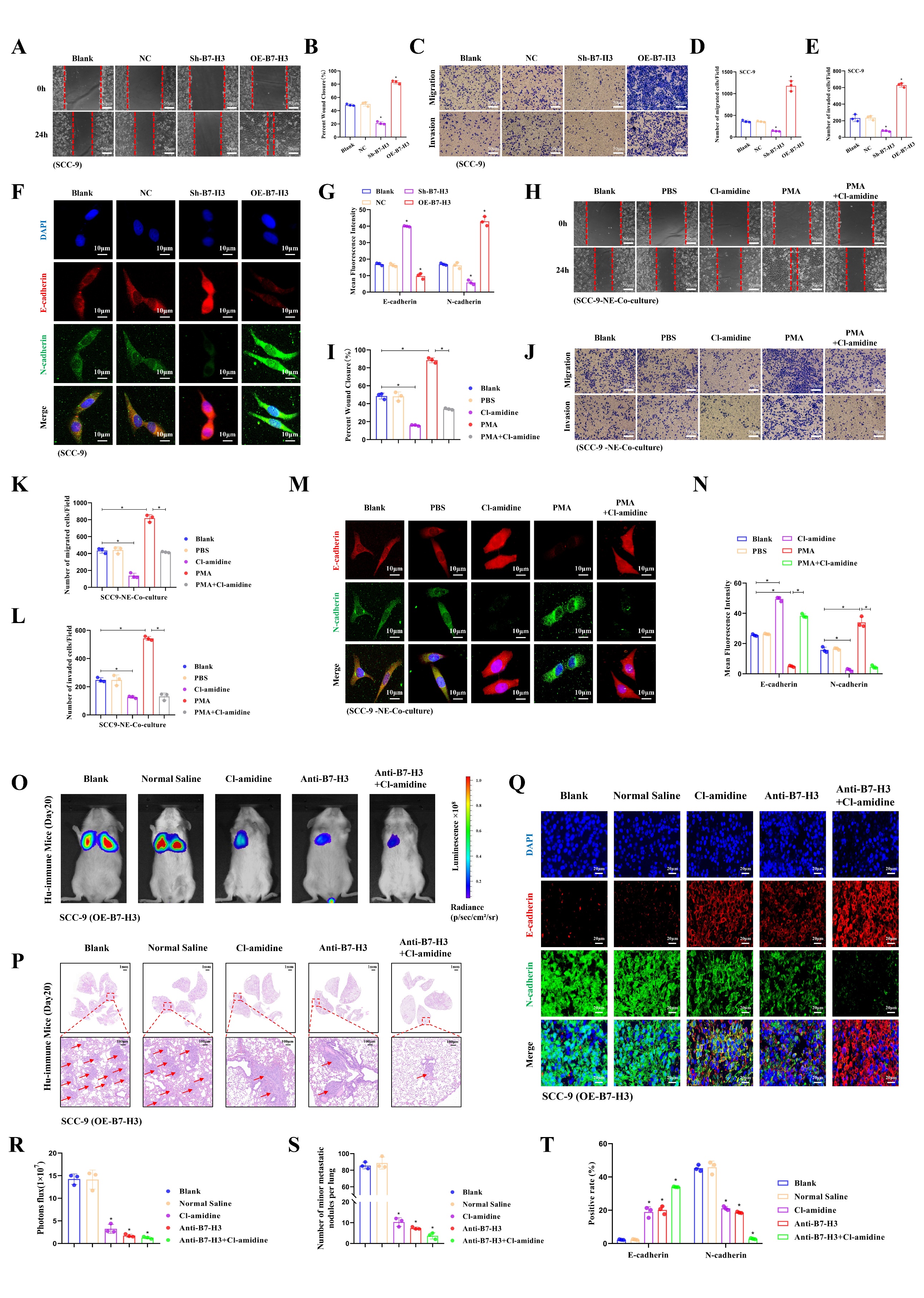


**Supplementary Figure 2. B7-H3 and NETs Promote OSCC Metastasis via the EMT Pathway In Vitro and In Vivo Validation in the SCC-9 Cell Line** (A) Wound-healing assay of SCC-9 cells with B7-H3 knockdown or overexpression (scale bars: 50 μm). (B) Statistical analysis of wound-healing assay results. (C) Transwell migration and invasion assays of SCC-9 cells with B7-H3 knockdown or overexpression (scale bars: 50 μm). (D) Statistical analysis of migration assay results. (E) Statistical analysis of invasion assay results. (F) IF staining for E-cadherin (red) and N-cadherin (green) in SCC-9 cells with B7-H3 knockdown or overexpression (scale bars: 10 μm). (G) Quantitative analysis of IF staining for E-cadherin and N-cadherin. (H) Wound-healing assay of SCC-9 cells (lower chamber) co-cultured with neutrophils (NE, upper chamber). Treatment groups: Blank, PBS, Cl-amidine, PMA, and PMA + Cl-amidine (scale bars: 50 μm). (I) Statistical analysis of wound-healing assay. (J) Transwell migration and invasion assays of SCC-9 cells (upper chamber) co-cultured with NE (lower chamber) (scale bars: 50 μm). (K) Statistical analysis of migration assay. (L) Statistical analysis of invasion assay. (M) IF staining for E-cadherin (red) and N-cadherin (green) in SCC-9 cells co-cultured with NE under different treatments (scale bars: 10 μm). (N) Quantitative analysis of IF staining for E-cadherin and N-cadherin in SCC-9 cells. (O) In vivo bioluminescence imaging of lung metastases in a mouse model injected with B7-H3-overexpressing SCC-9 cells at 20 days post-injection. (P) HE staining of metastatic lungs, with red arrows indicating metastatic foci (scale bars: 1 mm for low magnification/100 μm for high magnification). (Q) IF staining for E-cadherin (red), N-cadherin (green), and DAPI (blue) in metastatic tumors (scale bars: 20 μm). (R) Quantitative analysis of lung fluorescence intensity. (S) Quantitative analysis of lung metastatic foci. (T) Quantitative analysis of IF staining for E-cadherin and N-cadherin in metastatic tumors. n=3 (in vitro and in vivo experiments). Statistical methods: t-test for two groups, ANOVA-Tukey test for multiple-group comparisons. Data are presented as mean ± standard deviation (**p* < 0.05).


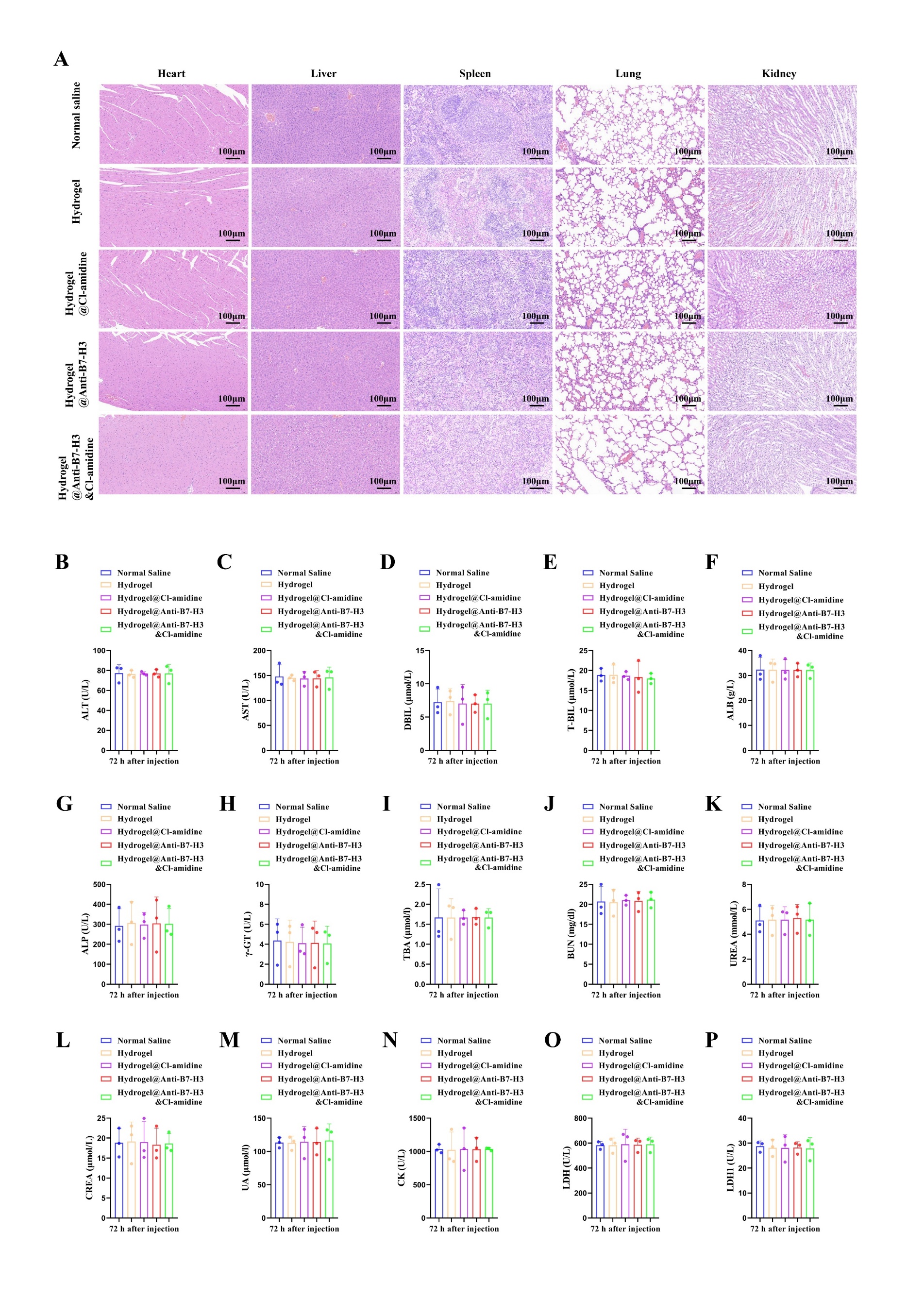


**Supplementary Figure 3. The safety evaluation of Hydrogel@Anti-B7-H3&Cl-amidine in vivo** (A) HE staining of heart, liver, spleen, lung, and kidney tissues from healthy mice in different treatment groups (Normal saline, Hydrogel, Hydrogel@Cl-amidine, Hydrogel@Anti-B7-H3, Hydrogel@Anti-B7-H3&Cl-amidine) (n=3, representative images) (scale bars: 100 μm). (B-P) Blood biochemical analysis of ALT, AST, DBIL, T-BIL, ALB, ALP, γ-GT, TBA, BUN, UREA, CREA, UA, CK, LDH, and LDH1 from healthy mice. Abbreviations: ALT: Alanine aminotransferase; AST: Aspartate aminotransferase; DBIL: Direct bilirubin; T-BIL: Total bilirubin; ALB: Albumin; ALP: Alkaline phosphatase; γ-GT: Gamma-glutamyl transferase; TBA: Total bile acid; BUN: Blood urea nitrogen; UREA: Urea; CREA: Creatinine; UA: Uric acid; CK: Creatine kinase; LDH: Lactate dehydrogenase; LDH1: Lactate dehydrogenase 1 (n=3, *p* > 0.05). ANOVA-Tukey test for multiple-group comparisons. Data are presented as mean ± SD, and *p* < 0.05 indicates statistically significant differences.


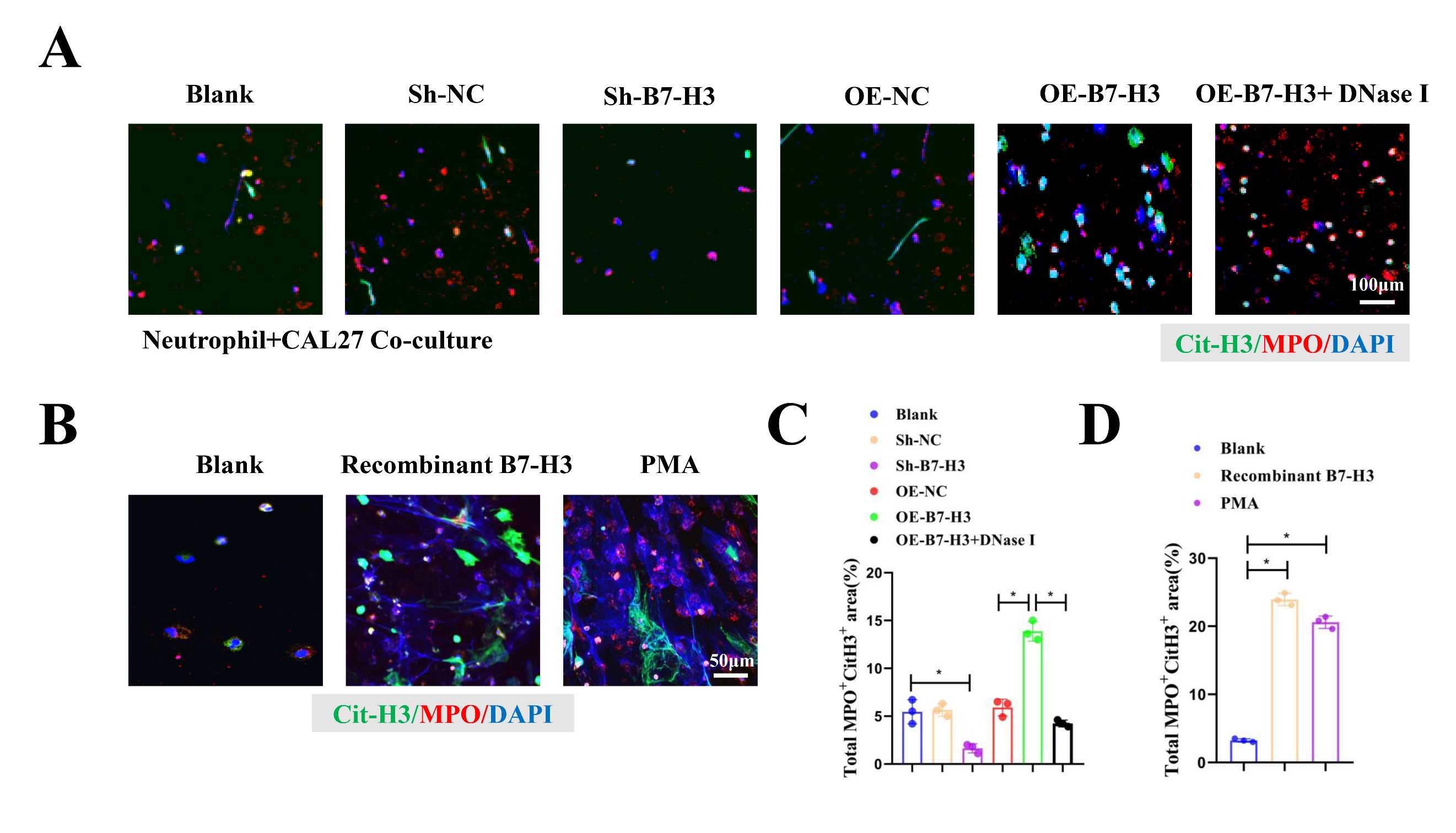


**Supplementary Figure 4. B7-H3 directly induces NETs formation in human neutrophils** (A) IF staining of Cit-H3 (green) and MPO (red) in neutrophils co-cultured with CAL-27 cells transfected with OE-B7-H3, Sh-B7-H3, or respective controls. Scale bar: 100 μm. (B) IF staining of Cit-H3 (green) and MPO (red) in neutrophils treated with recombinant B7-H3 (0.5 μg/mL) or PMA (100 nM). Scale bar: 50 μm. (C) Quantitative analysis of NETs in (A). (D) Quantitative analysis of NETs in (B). Experiments were repeated three times independently. Statistical analyses: unpaired t-test (two groups) and one-way ANOVA with Tukey’s post hoc test (multiple groups). Data are mean ± SD (**p* < 0.05).


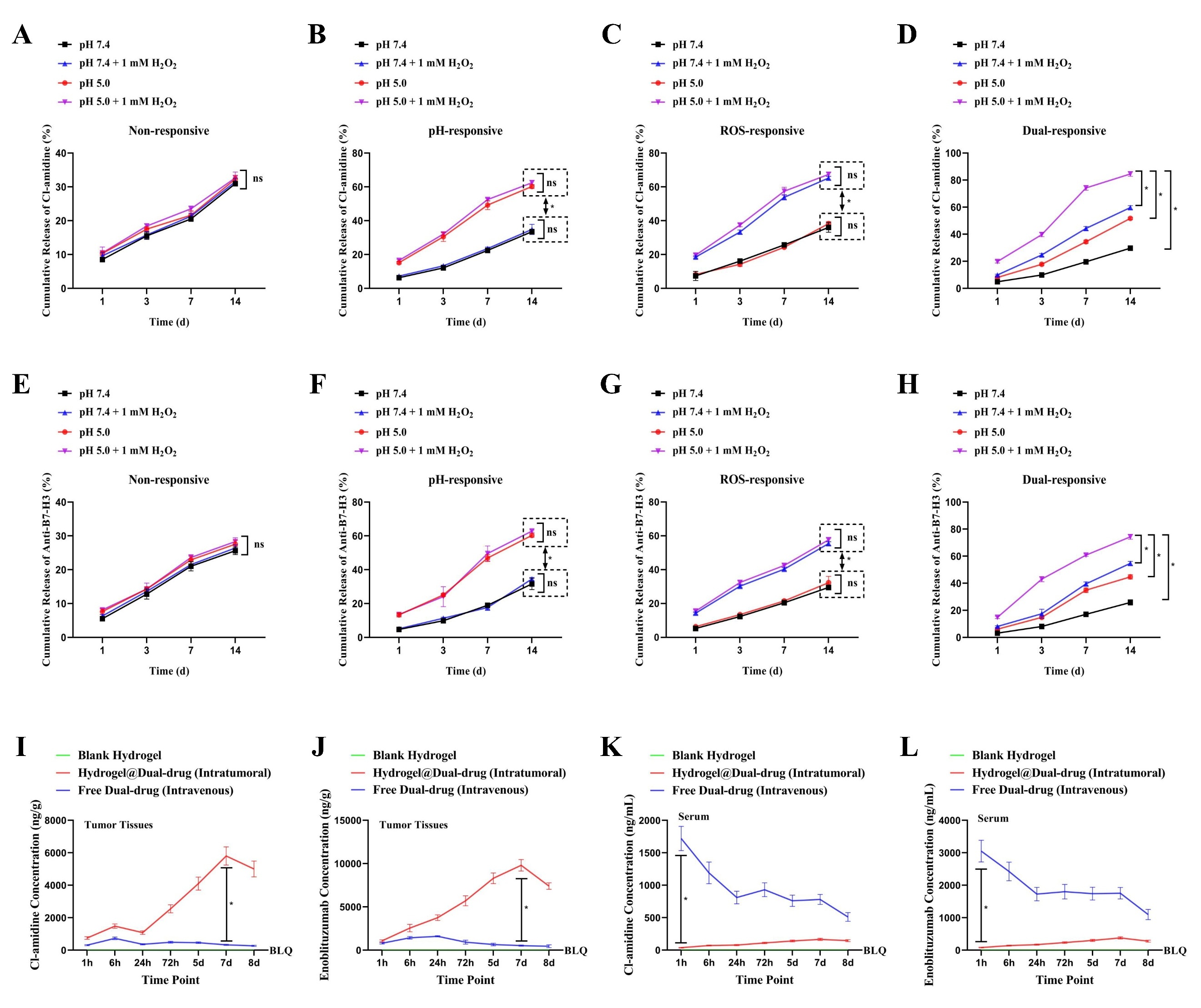


**Supplementary Figure 5. In Vitro Drug Release Profiles of Hydrogel and Its In Vivo Tumor-Serum Pharmacokinetics** (A-D) Cumulative release profiles of Cl-amidine from four hydrogel systems (non-responsive hydrogel, pH-responsive hydrogel, ROS-responsive hydrogel, and dual-responsive hydrogel) across media with varying pH and H₂O₂ conditions. (E-H) Corresponding cumulative release profiles of Enoblituzumab from these four hydrogels under the same conditions. (I-L) Pharmacokinetic profiles: (I) Cl-amidine levels in tumor tissue, (J) Enoblituzumab levels in tumor tissue, (K) Cl-amidine levels in serum, and (L) Enoblituzumab levels in serum. All experiments were run in triplicate. Statistical analyses used unpaired t-tests (for two-group) and one-way ANOVA with Tukey’s post hoc tests (for multiple-group). Data are reported as mean ± SD, with **p* < 0.05 denoting statistical significance.


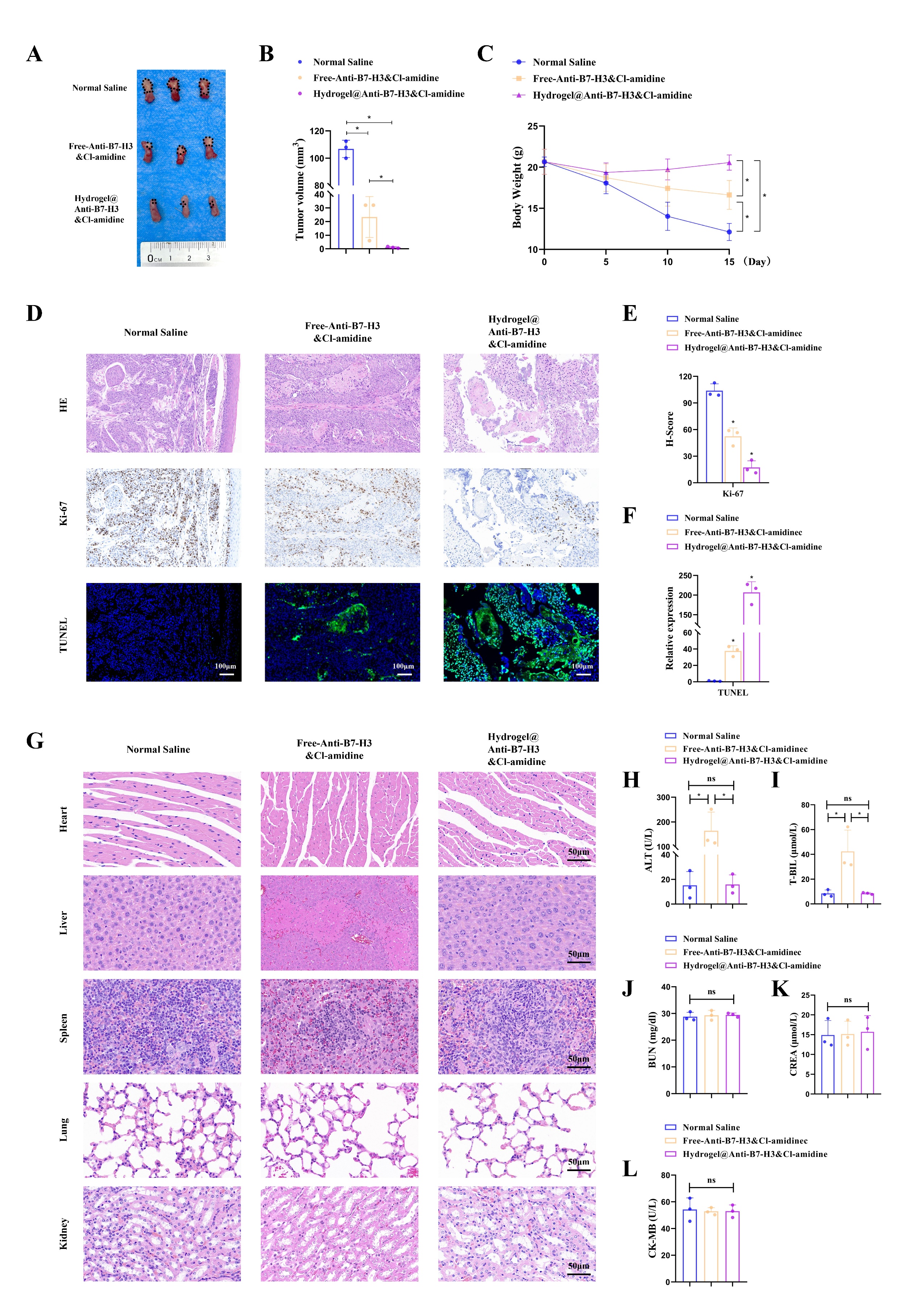


**Supplemental Figure 6: Free drug control experiment: Validating pH/ROS-responsive hydrogel’s efficacy and biosafety superiority compared to free dual drugs.** (A) Representative photographs of tongue tumors harvested from orthotopic OSCC mice in each group (Normal Saline, Free-Anti-B7-H3&Cl-amidine, Hydrogel@Anti-B7-H3&Cl-amidine; all via local injection). (B) Quantitative analysis of tumor volume. (C) Dynamic changes in body weight of mice. (D) HE staining, Ki-67, and TUNEL staining of tumor tissues in each group (scale bar: 100 μm). (E) Quantitative analysis of Ki67-positive rate. (F) Quantitative analysis of TUNEL-positive rate. (G) HE staining of major organs (heart, liver, spleen, lung, kidney) from mice in each group (scale bar: 50 μm). (H-L) Quantitative analysis of blood biochemical indices: ALT, T-BIL, BUN, CREA, CK-MB. Experiments were repeated three times independently. Statistical analyses: one-way ANOVA with Tukey’s post hoc test. Data are mean ± SD (**p* < 0.05). The dosage and administration schedule of the free drugs were exactly the same as those of the Hydrogel@Anti-B7-H3&Cl-amidine group.


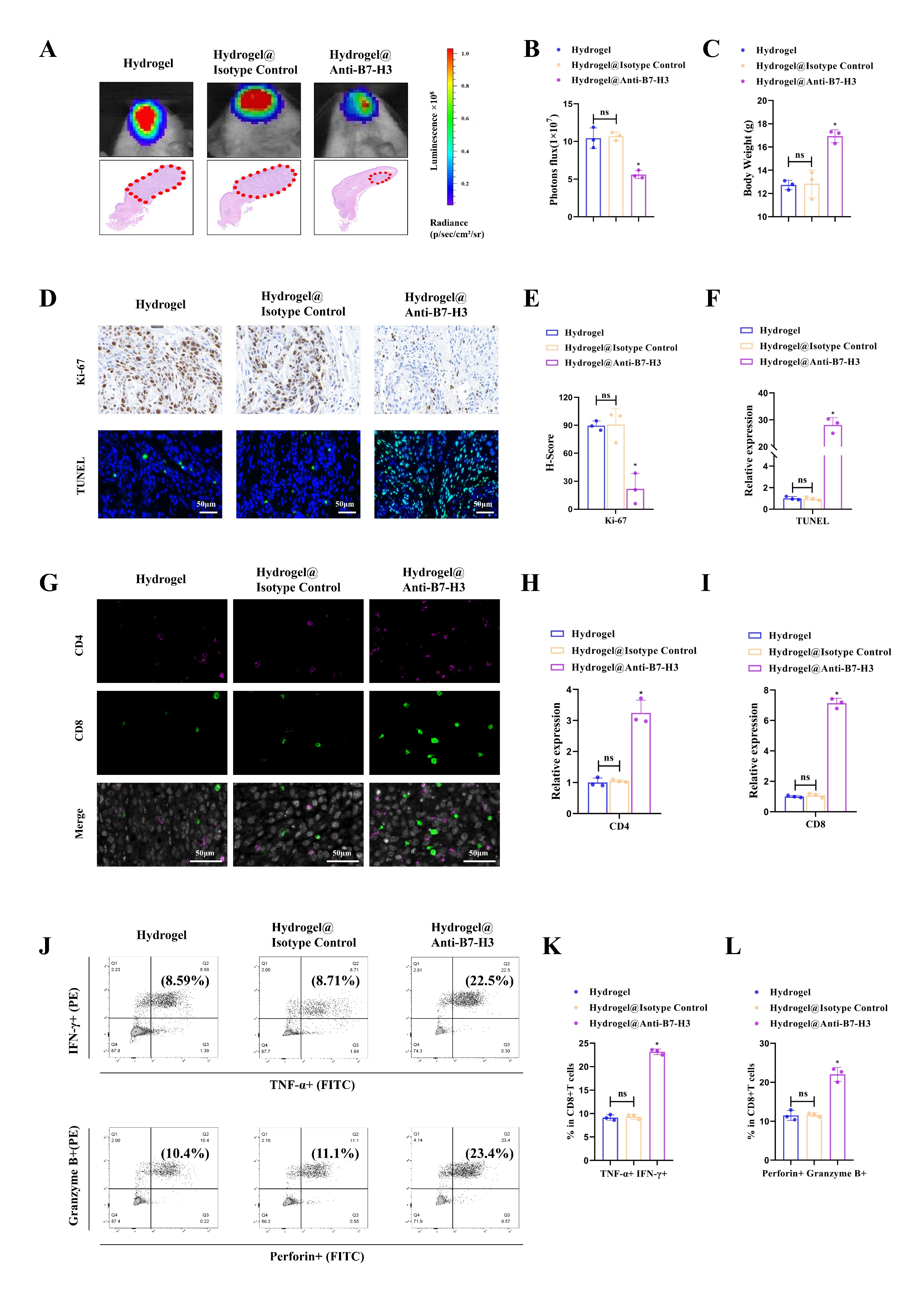


**Supplementary Figure 7. Isotype Control Validates Specific Antitumor Efficacy of Hydrogel@Anti-B7-H3.** (A) In vivo bioluminescence imaging and HE staining of orthotopic tongue tumors in three treatment groups: Blank Hydrogel, Human IgG1 kappa Isotype Control-loaded Hydrogel, Anti-B7-H3 Hydrogel group. (B) Quantitative analysis of bioluminescence intensity from in vivo imaging (n=3). (C) Body weight of mice in each group. (D) Representative images of Ki-67 IHC staining and TUNEL IF staining in tumor tissues. (E) Quantitative analysis of Ki-67 in tumor tissues. (F) Quantitative analysis of TUNEL in tumor tissues. (G) Representative images of IF staining for CD4⁺ T cells (purple) and CD8⁺ T cells (green) in tumor tissues. (H) Quantitative analysis of CD4⁺ T cells. (I) Quantitative analysis of CD8⁺ T cells. (J-L) Flow cytometry showing the expression of TNF-α, IFN-γ, Perforin, and Granzyme B in tumor-infiltrating CD8⁺ T cells. Statistical analyses: one-way ANOVA with Tukey’s post hoc test. Data are mean ± SD, ns (not significant), **p* < 0.05.


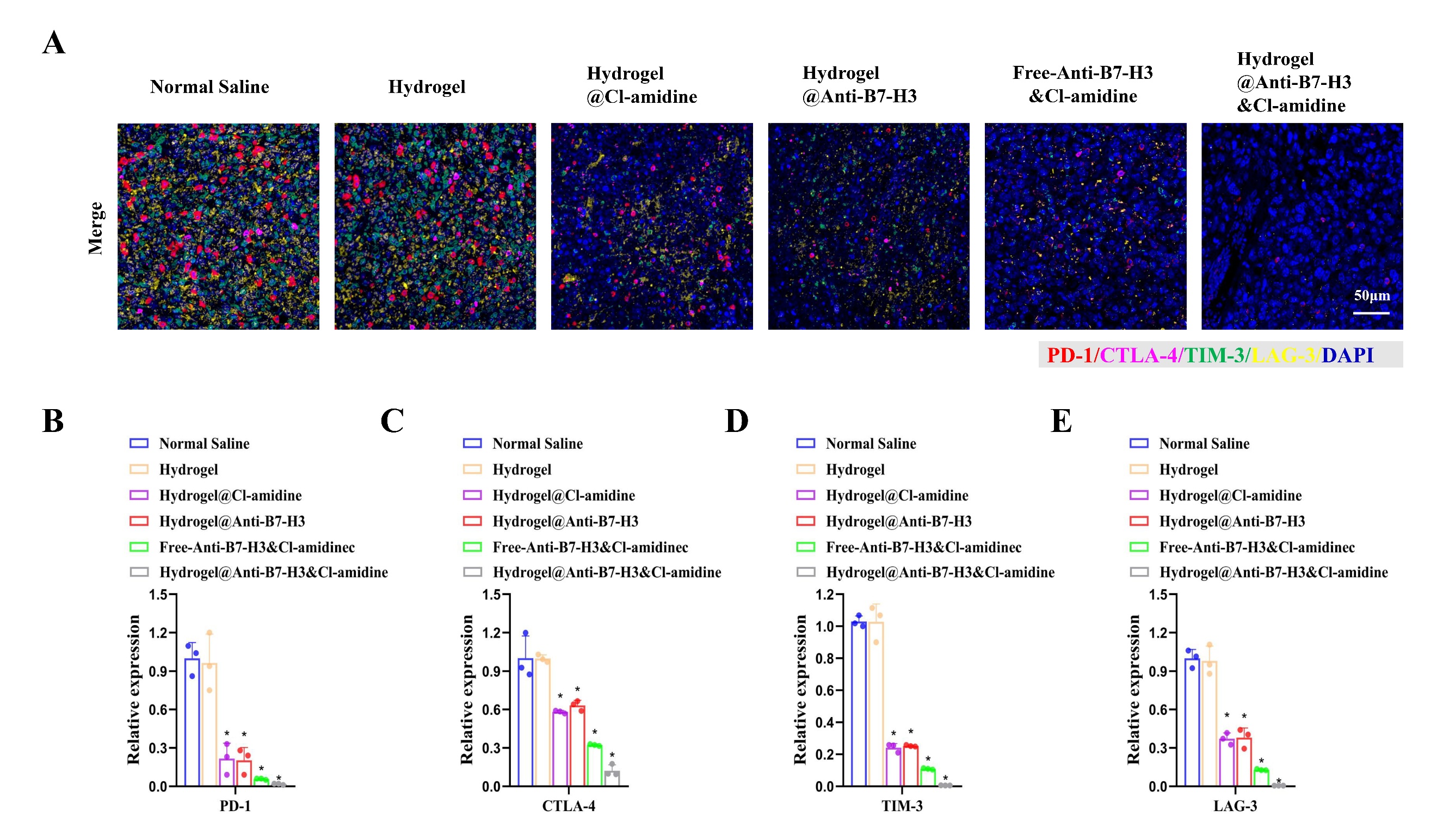


**Supplementary Figure 8. Expression of Inhibitory Checkpoint Molecules (PD-1, CTLA-4, TIM-3, LAG-3) in Subcutaneous OSCC Tumor Tissues.** (A) Representative IF images of inhibitory checkpoint molecules in tumor tissue sections (scale bar: 50 μm). PD-1 (red), CTLA-4 (pink), TIM-3 (green), LAG-3 (yellow), and DAPI (blue, nuclear staining). (B-E) Quantitative analysis of PD-1 (B), CTLA-4 (C), TIM-3 (D), and LAG-3 (E) positive rates. Experiments were repeated three times independently. Statistical analyses: one-way ANOVA with Tukey’s post hoc test (multiple groups). Data are mean ± SD (**p* < 0.05).


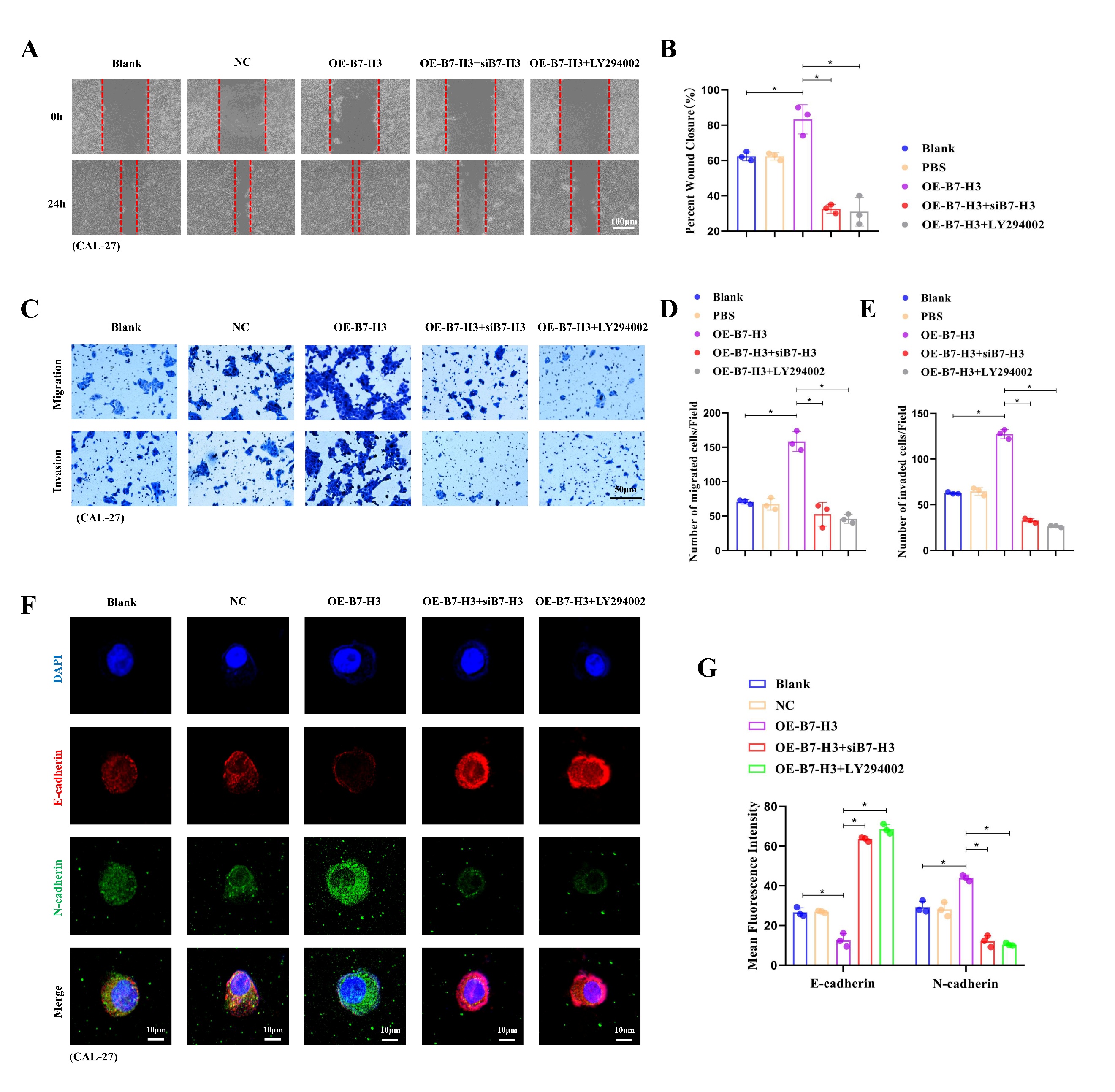


**Supplementary Figure 9. B7-H3-induced invasion, migration and EMT in CAL-27 cells reversed by B7-H3 siRNA or LY294002.** (A) Wound healing assay of CAL-27 cells in indicated groups. (B) Quantitative analysis of wound closure rate in (A). (C) Transwell migration and invasion assays of CAL-27 cells in indicated groups. (D-E) Quantitative analysis of migrated (D) and invaded (E) cell numbers in (C). (F) IF staining of E-cadherin (red) and N-cadherin (green) in CAL-27 cells (DAPI: blue). (G) Quantitative analysis of mean fluorescence intensity of E-cadherin and N-cadherin in (F). Groups: OE-B7-H3, OE-B7-H3+siB7-H3, OE-B7-H3+LY294002 (PI3K inhibitor); Blank and NC groups as controls. Experiments were repeated three times independently. Statistical analyses: one-way ANOVA with Tukey’s post hoc test. Data are mean ± SD (**p* < 0.05).
